# Select early growth response (Egr) isoforms augment hypoxia inducible factor 2 (HIF-2) regulation of erythropoietin (Epo) gene expression in mammals

**DOI:** 10.1016/j.jbc.2025.110355

**Published:** 2025-06-10

**Authors:** Jason S. Nagati, Elhadji M. Dioum, Catarina S. Giardinetto, S.M. Golam Mohiuddin, Qiuyang Zhang, Joseph A. Garcia

**Affiliations:** 1 Department of Medicine, Columbia University Medical Center, New York, New York, USA; 2 Department of Medicine, University of Texas Southwestern Medical Center, Dallas, Texas, USA; 3 Department of Research, James J. Peters VA Medical Center, Bronx, New York, USA

**Keywords:** erythropoietin, epo, hypoxia-inducible factor (HIF), HIF-1, HIF-2, early growth response (Egr), Egr1, Egr2, Egr3, neural crest, Hep3B

## Abstract

Hypoxia inducible factors (HIFs) are heterodimeric, oxygen-sensitive, stress-responsive transcription factors in vertebrates composed of one of three alpha components conferring oxygen sensitivity and one of four beta components required for dimerization and DNA binding. The founding alpha member, HIF-1α, is ubiquitously expressed and regulates numerous target genes required for cellular function under physiological hypoxic states. In comparison, HIF-2α is more restricted in its expression, but nevertheless is also required for normal physiological function. The prototypical HIF-2 target gene is erythropoietin (*Epo*), one of the most highly hypoxia-inducible genes in mammals and in Hep3B cells, a model cell line used to study hypoxia-inducible gene regulation. However, despite cell culture and *in vivo* data supporting *Epo* as a preferential HIF-2 target, the molecular basis for selective activation of Epo by HIF-2 remains unknown. In this study, we report identification of novel evolutionary conserved *cis*-acting elements in the mammalian 3′ *Epo* enhancer region that includes recognition sites for stress-responsive early growth response (Egr) transcription factors, demonstrate that select Egr2 and Egr3 isoforms augment HIF-2 activation of a reporter containing the extended 3′ *Epo* enhancer with Egr binding sites, reveal stable Egr2/HIF-2 complex formation in hypoxic Hep3B cells, and provide conditional knockout data from mice supporting an *in vivo* role for Egr2 in *Epo* gene regulation. These results provide insights into HIF-2 selective signaling mechanisms with ramifications that extend well beyond *Epo* regulation.

Erythropoietin (Epo), a proerythrogenic hormone produced in fetal liver and adult kidney, regulates erythropoiesis in mammals (1, 2, 3, 4, 5). Adult animals subjected to systemic hypoxia or severe anemia exhibit marked increases in serum Epo protein, predominantly due to increased renal and hepatic *Epo* mRNA synthesis (6). Comparison of human and mouse *Epo* genes identified candidate hypoxia responsive regions including one in the 3′-flanking sequence (7, 8, 9, 10). The use of two hepatoma cell lines, Hep3B and HepG2, that exhibit hypoxia-induced *Epo* gene expression facilitated isolation of the hypoxia-responsive *Epo* 3′ enhancer (10, 11, 12). The *Epo* 3′ enhancer hypoxia responsive region was further demarcated to a minimal 43-basepair or 50-basepair hypoxia responsive motif (9, 13), also induced by the chemical hypoxia mimetic cobalt chloride (14, 15). Using this as a molecular handle, hypoxia inducible factor 1 (HIF-1), a master transcriptional regulator of hypoxic and other environmental stress responses (16, 17), was biochemically purified and its subunits identified.

Studies of transgenic mouse models confirmed a role for HIF-1 in *Epo* regulation during early development. During early mouse embryogenesis, primitive erythropoiesis localizes to the extraembryonic yolk sac (18). *HIF-1*α^−/−^ embryos, which expire by E10.5 (19, 20, 21), have reduced *Epo* gene expression and yolk sac hematopoiesis at E9.5 (22), consistent with impaired primitive erythropoiesis due to a loss of HIF-1α dependent regulation of *Epo* gene expression. Around embryonic day 9.5 (E9.5), Epo production starts in the fetal liver blood islands (23), which become the major source of red blood cells as well as *Epo* production in the developing embryo by E14.5 (24, 25). Epo is also expressed in early hematopoietic precursor/progenitor cells (26).

With completion of the human genome project, a second HIF alpha member was identified, endothelial PAS domain protein 1 (*EPAS1*) also known as HIF-2α, HLF, HRF, or MOP2 (27, 28, 29, 30), which regulates key physiological processes in mammals. The transition to definitive erythropoiesis starts ∼ E12, just prior to when *HIF-2*α^*−/−*^ (31) as well as *Epo*^−/−^ (32) knockout mice expire. Consistent with a role for HIF-2 in late development, E14.5 *HIF-2*α^*−/−*^ mice demonstrate markedly reduced hepatic *Epo* mRNA levels (33).

Using a genetic breeding strategy, viable *HIF-2*α^*−/−*^ mice were generated and characterized (33, 34). *HIF-2*α^*−/−*^ mice display multilineage hematopoietic defects (35) and marked reductions in renal *Epo* mRNA levels (33). Adult *HIF-2*α^*+/−*^ mice lack gross anatomical abnormalities, but have blunted renal *Epo* expression following systemic hypoxic exposure (33). Subsequent conditional knockout mice studies later confirmed that HIF-2α and not HIF-1α regulates *Epo* expression in adult liver (36). Cell culture studies using RNA interference also found that HIF-2α regulates *Epo* gene expression in Hep3B cells and other cell lines that produce Epo protein in a hypoxia-regulated manner (37). These cell culture and *in vivo* mouse data support HIF-2α as an essential HIF alpha member regulating Epo production in Epo-inducible cell lines and the primary HIF alpha member regulating endocrine Epo production in adult mammals.

HIF-1α is stabilized during hypoxia, forms a heterodimer with a HIF beta partner, and the HIF-1 heterodimer binds to regulatory elements in target genes *via* a conserved DNA motif known as the HIF-responsive element (HRE). The HIF-2 heterodimer likewise binds HREs and induces expression of reporter genes containing isolated HREs found in the 3′ *Epo* enhancer region and other hypoxia or HIF-1 responsive genes (27, 28). Although ectopic expression of HIF-1α and HIF-2α both augment induction of the 3′ minimal *Epo* enhancer region in isolated reporter assays, we hypothesized that the minimal *Epo* enhancer lacks *cis*-elements important for preferential HIF-2α activation.

## Results

**Figure 1:**
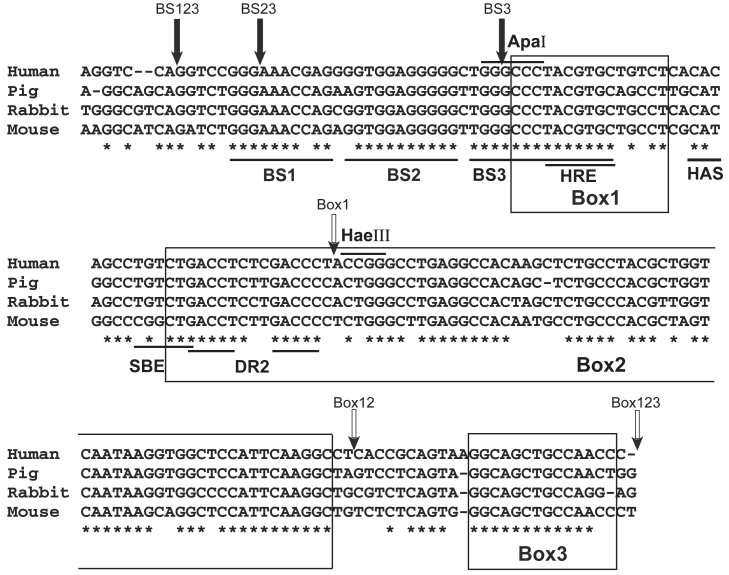
**Evolutionary conserved elements in the mammalian 3′ *Ep*o enhancer**. Sequence alignment of mammalian *Epo* enhancer. Box 1 contains the previously identified HIF binding hypoxia responsive element (HRE) with its core sequence (ACGTG) underlined. Two additional regions of extensive identity are designated Box 2 and Box 3. The HIF accessory sequence (HAS), which resides between *boxes 1* and *2*, is CAC in humans and CAT in mice. DR2 in *box 2* is a dyad repeat and putative HNF-3 binding site. Additional homology regions located 5′ of the HRE are labeled BS1, BS2, and BS3. *Solid arrows* indicate 5′ end of BS123, BS23, and BS3 constructs, and *open arrows* indicate 3′ end of the Box1, Box12, and Box123 constructs. BS, binding site; Epo, erythropoietin; HIF, hypoxia inducible factor.

To identify evolutionary conserved *cis*-elements in the *Epo* enhancer region, we aligned several mammalian *Epo* enhancer regions using rVISTA 2.0 (38) and manual inspection (Fig. 1). We identified three conserved large blocks in the 3′ *Epo* enhancer, designated boxes 1 through 3. The 5′-most Box 1 contains the HRE whereas the 5′ portion of Box 2 contains a direct repeat of two tandem nuclear steroid receptor half-sites (DR-2), which were postulated as functional elements of the minimal 3′ Epo enhancer region (39). Manual and BLAST inspection identified additional conserved smaller sequences 5′ to Box 1, which we designated binding sites (BSs) 1 through 3 with BS3 encompassing the HRE, thus overlapping Box 1.

**Figure 2:**
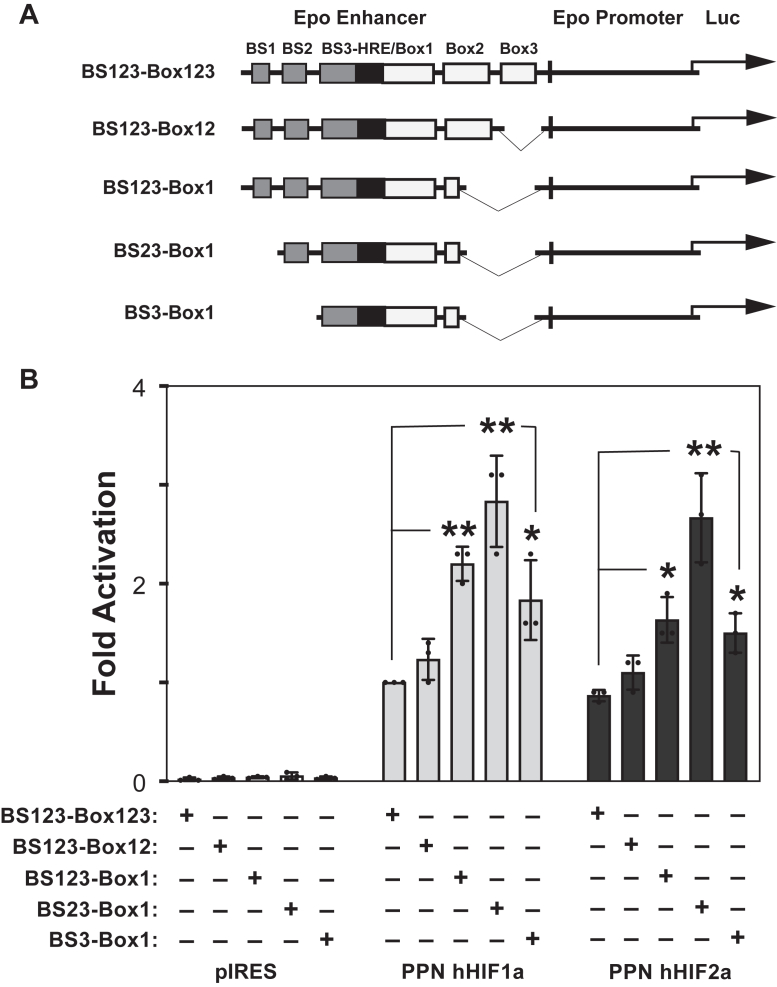
**Conserved sequences in the 3′ *Epo* enhancer modulate HIF-dependent activation**. *A*, mouse 3′ *Epo* enhancer regions retaining or lacking putative control elements fused to the mouse *Epo* promoter and a luciferase reporter. *B*, HEK293T cells cotransfected with the indicated mouse *Epo* enhancer-promoter reporter plasmids along with empty expression vector or plasmids encoding mutant (PPN) HIF-1α or PPN HIF-2α rendered oxygen-insensitive by substitution mutations for the two prolines (PP) and one asparagine (N) modified by oxygen-dependent prolyl or asparaginyl hydroxylases. Data shown are the mean and standard deviation of three independent sets of transfections with each sample for each set assessed in triplicate transfections. Comparison of reporter activation was assessed by ordinary one-way ANOVA with two families, PPN HIF-1α or PPN HIF-2α, followed by Dunnett's multiple comparisons test with adjusted *p* values reported. Significant increases relative to BS123-Box123 were noted for BS123-Box1 (*p* = 0.0021), BS23-Box1 (*p* < 0.0001), and BoxBS3-Box1 (*p* = 0.0217), but not Box123-Box12, by PPN HIF-1α. Significant increases relative to BS123-Box123 were also noted for BS123-Box1 (*p* = 0.0144), BS23-Box1 (*p* < 0.0001), and BoxBS3-Box1 (*p* = 0.0405), but not Box123-Box12, by PPN HIF-2α. Comparisons were also made for BS23-Box1 to BS2-Box1 using Welch's *t* test. Significant reductions were noted for activation of BS3-Box1 relative to BS23-Box1 by PPN HIF-1α (*p* = 0.0487) or PPN HIF-2α (*p* = 0.0309). ∗*p* ≤ 0.05, ∗∗*p* ≤ 0.01. BS, binding site; Epo, erythropoietin; HIF, hypoxia inducible factor

We defined the contribution of the conserved elements to *Epo* regulation and HIF activation using deletion mutagenesis in conjunction with transient transfected reporter assays (Fig. 2). We generated deletion constructs of the extended parental mouse 3′ *Epo* enhancer region (BS123-Box123) and fused these to the mouse *Epo* promoter placed upstream of firefly luciferase (Fig. 2*A*). We assessed basal expression and HIF-inducibility of these heterologous reporters in HEK293T cells using mutant (PPN) HIF-1α or HIF-2α rendered oxygen-insensitive with substitution mutations for the two prolines (PP) and one asparagine (N) modified by oxygen-dependent prolyl or asparaginyl hydroxylases (Fig. 2*B*). We examined 3′ deletion constructs that lack either Box 3 (BS123-Box12), or Box 2 and 3 (BS123-Box1) and 5′ deletion constructs of BS123-Box1 that lack either BS1 (BS23-Box1) or BS1 and 2 (BS3-Box1). Boxes 2 and 3 have an inhibitory effect upon HIF activation as their deletion (BS123-Box123 *versus* BS123-Box1) resulted in a significant increase in activation by both PPN HIF-1α (*p* = 0.0021) and PPN HIF-2α (*p* = 0.0144). Deletion of BS1 (BS123-Box1 *versus* BS23-Box1) further augments activation by PPN HIF-1α (*p* < 0.0001) or PPN HIF-2α (*p* < 0.0001) and resulted in maximal activity of the isolated reporter. Eliminating BS2 (BS23-Box1 *versus* BS3-Box1) resulted in attenuated induction of the isolated reporter by PPN HIF-1α (*p* = 0.0217) or PPN HIF-2α (*p* = 0.0405). Of note, the BS3-Box1 construct is nearly identical to the minimal human *Epo* enhancer region delineated by an ApaI/HaeIII restriction endonuclease fragment (14).

**Figure 3:**
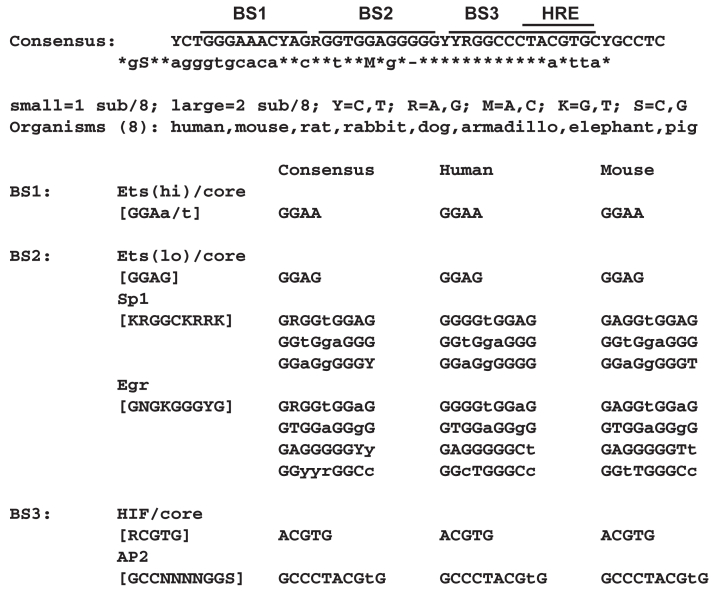
**Candidate transcription factor recognition sites in the 3′ *Ep*o enhancer**. Predicted transcription factor binding sites for BS1, BS2, and BS3 based on consensus binding sequences including the previously identified HIF binding site HRE residing in BS3. BS2 contains a consensus binding site for the EGR transcription factor family. EGR, early growth response; Epo, erythropoietin; HRE, HIF-responsive element; HIF, hypoxia inducible factor.

We hypothesized that BS2 may serve as a DNA binding site for transcription factors that act in synergy with HIF-1 and/or HIF-2. Bioinformatic analyses (40) indicated BS2, a GC-rich sequence, is a consensus recognition site for early growth response (Egr) transcription factors (Fig. 3). The Egr family consists of four members that share similar DNA binding domains, but differ in other regions: Egr1, also called ZIF268, NGFI-A (nerve growth factor inducible gene A), Krox24, and TIS8 (41, 42, 43); Egr2, also known as Krox20 (44); Egr3, also known as PILOT (45); Egr4, also known as NGFI-C (46). Egr1-3 contain a domain known as R1 that mediates interactions with the Nab1 and Nab2 proteins (47, 48), which in many, but not all (49, 50), cases represses the transactivation capacity of Egr1-3 proteins (47, 48, 51, 52). Egr1-3 induces expression of Nab2, which may function as a negative feedback loop (53). Egr4 does not contain an R1 domain and is insensitive to Nab protein action.

**Figure 4:**
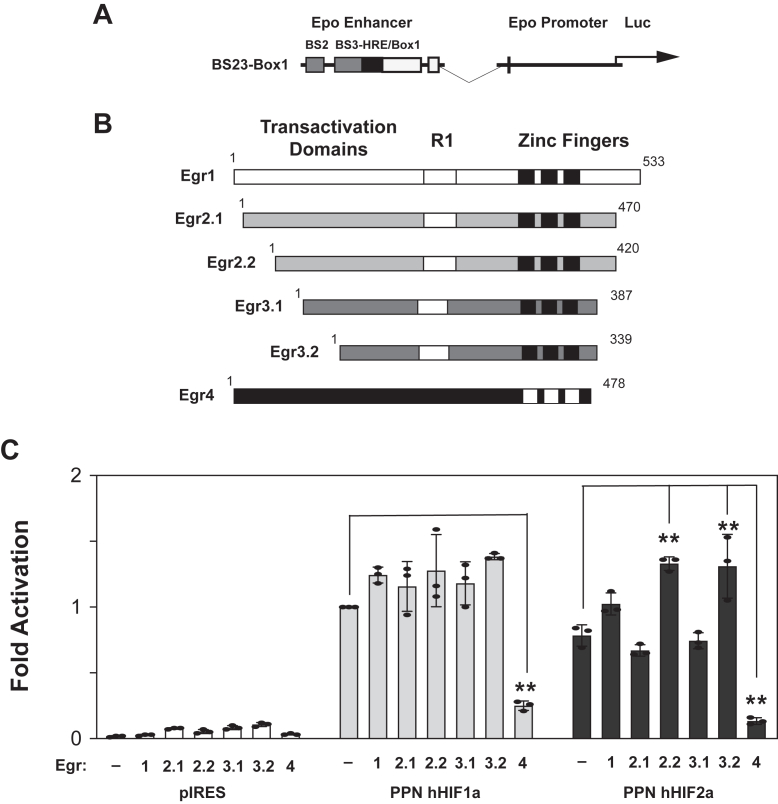
**Select Egr members augment HIF activation of a 3′ *Epo* enhancer reporter**. *A*, the mouse 3′ *Epo* enhancer BS23-Box1 reporter containing a putative Egr binding site in BS2. *B*, schematic representation of parental Egr family members. *C*, HEK293T cells cotransfected with the mouse 3′ *Epo* enhancer BS23-Box1 reporter along with expression plasmids encoding mutant oxygen-insensitive PPN HIF-1α or PPN HIF-2α with control or Egr expression plasmids. Data shown are the mean and standard deviation of three independent sets of transfections with each sample for each set assessed in triplicate. Comparison of reporter activation between Egr proteins was assessed by ordinary one-way ANOVA with two families, PPN HIF-1α or PPN HIF-2α, followed by Tukey's multiple comparisons test with adjusted *p* values reported for comparisons to control determinations. For PPN HIF-1α, none of the Egr proteins augmented reporter activity. For PPN HIF-2α, significant augmentation was noted with the shorter Egr2 (Egr2.2, *p* = 0.0004) and Egr3 (Egr3.2, *p* = 0.0005) isoforms. There was significant repression by Egr4 with both PPN HIF-1α (*p* = 0.0003) and PPN HIF-2α (*p* < 0.0001). ∗∗*p* ≤ 0.01. BS, binding site; Egr, early growth response; Epo, erythropoietin; HIF, hypoxia inducible factor.

To assess the role of Egr members in HIF activation of the *Epo* enhancer reporter, we performed transient transfection reporter assays (Fig. 4). Because Egr2 and Egr3 are alternatively spliced in mice and humans, we examined two isoforms each for Egr2 and Egr3 that differ in length of their amino terminal activation domains (Fig. 4*B*). We cotransfected Egr1-4 expression plasmids along with PPN HIF-1α or PPN HIF-2α expression plasmids and measured induction of the wildtype (WT) BS23-Box1 *Epo* enhancer reporter in HEK293T cells (Fig. 4*C*). For PPN HIF-1α, none of the Egr proteins augmented reporter activity. For PPN HIF-2α, there was significant augmentation by the shorter Egr2 isoform, Egr2.2, (*p* = 0.0004) and the shorter Egr3 isoform, Egr3.2, (*p* = 0.0005). Egr4 repressed activation by both PPN HIF-1α (*p* < 0.0001) and PPN HIF-2α (*p* < 0.0001).

**Figure 5:**
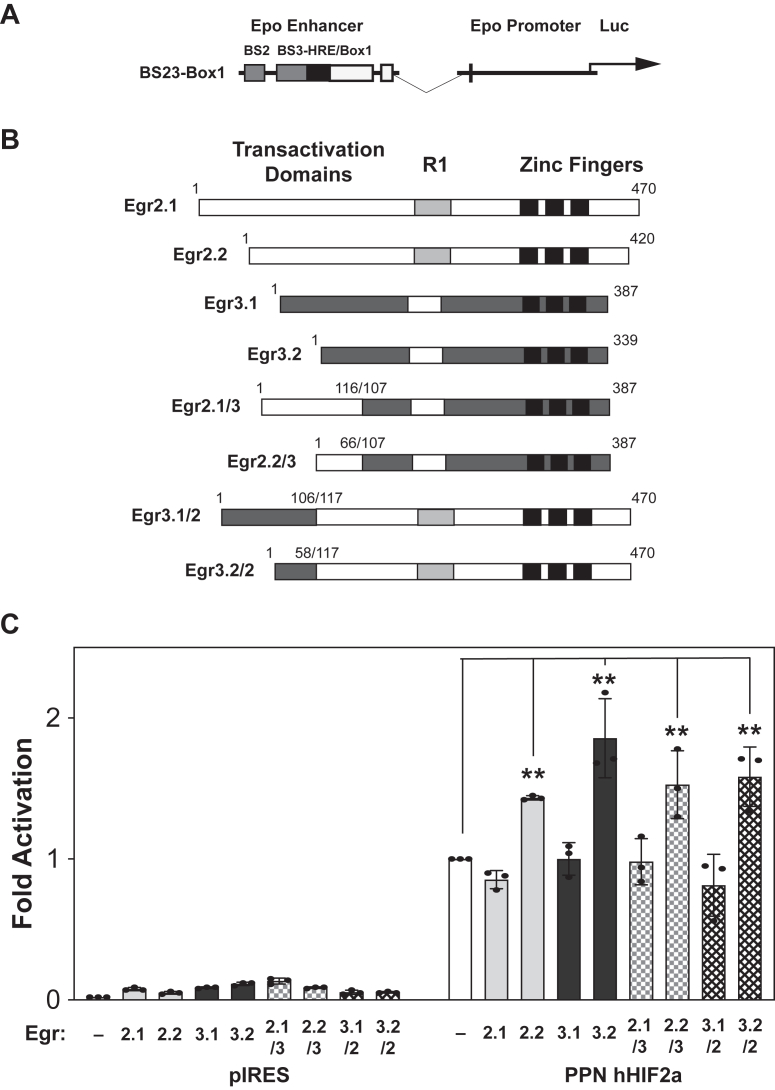
**The Egr2 and Egr3 isoform amino terminus controls its ability to synergize with HIF2**. *A*, the mouse 3′ *Epo* enhancer BS23-Box1 reporter containing a putative Egr binding site in BS2. *B*, schematic representation of Egr2 and Egr3 parental and hybrid constructs. *C*, HEK293T cells cotransfected with the mouse 3′ *Epo* enhancer BS23-Box1 reporter and an expression plasmid encoding mutant oxygen-insensitive PPN HIF-2α along with control or Egr expression plasmids. Data shown are the mean and standard deviation of three independent sets of transfections with each sample for each set assessed in triplicate. Comparison of reporter activation between Egr parental and hybrid proteins sharing the same amino termini was assessed by ordinary one-way ANOVA with two families, Egr2 and Egr3, followed by Tukey's multiple comparisons test to adjust *p* values. We confirmed synergistic effects for the shorter parental Egr2 (Egr2.1 *versus* Egr2.2, *p* = 0.0057) and Egr3 (Egr3.1 *versus* Egr3.2, *p* < 0.0001) proteins. Hybrids containing the amino terminus of the shorter Egr2 (Egr2.1 *versus* Egr2.2/3, *p* = 0.0013) or Egr3 (Egr3.1 *versus* Egr3.2/2, *p* = 0.0054) isoform also exhibited synergy with PPN HIF-2α. In contrast and like the longer parental isoforms, fusions with the longer Egr2 (Egr2.1/3) or Egr3 (Egr3.1/2) amino terminus did not augment activation. Similar conclusions were drawn when comparing all samples to control (PPN HIF-2α alone) as assessed by ordinary one-way ANOVA followed by Dunnett's multiple comparisons test. ∗∗*p* ≤ 0.01. BS, binding site; Egr, early growth response; Epo, erythropoietin; HIF, hypoxia inducible factor.

Because the longer Egr2 and Egr3 isoforms do not augment transactivation of the 3′ *Epo* enhancer reporter by PPN HIF-2α, in contrast to the shorter isoforms, we posited that the amino terminal portions of Egr2 and Egr3 dictate activation potential for the Epo enhancer region in conjunction with PPN HIF-2α (Fig. 5). We generated hybrid proteins that replaced proximal amino terminal portions of short and long Egr2 isoforms with corresponding regions from Egr3, or *vice versa* (Fig. 5*B*). We examined the transactivation capacity of these fusion proteins in comparison to the parental short and long isoforms (Fig. 5*C*). We confirmed synergistic effects for the shorter parental Egr2 (Egr2.1 *versus* Egr2.2, *p* = 0.0057) and Egr3 (Egr3.1 *versus* Egr3.2, *p* < 0.0001) proteins. Hybrids containing the amino termini of the shorter Egr2 (Egr2.1 *versus* Egr2.2/3, *p* = 0.0013) or Egr3 (Egr3.1 *versus* Egr3.2/2, *p* = 0.0054) isoforms also exhibited synergy with PPN HIF-2α. In contrast, fusions with the longer Egr2 (Egr2.1 *versus* Egr2.1/3) or Egr3 (Egr3.1 *versus* Egr3.1/2) amino terminus were ineffective at augmenting activation like the parental longer isoform protein.

**Figure 6:**
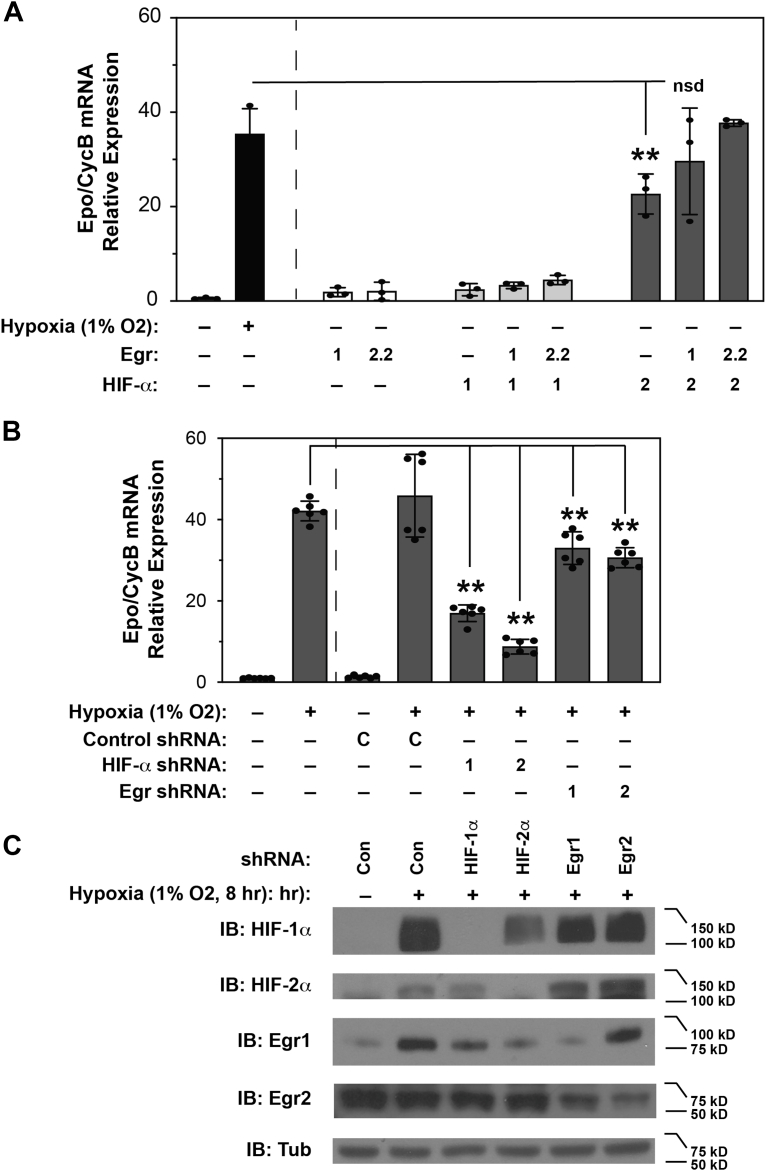
**Egr signaling regulates Epo induction in Hep3B cells**. *A*, comparison of relative *Epo* gene induction by hypoxia to induction under normoxia by mutant oxygen-insensitive PPN HIF-1α or PPN HIF-2α coexpressed with control (pIRES), Egr1, or Egr2 in Hep3B cells. Data shown are the mean and standard deviation of three transfections performed for each sample with each transfection assessed in triplicate. Comparison of transfected cells to untransfected Hep3B cells exposed to normoxia or hypoxia was assessed by ordinary one-way ANOVA followed by Dunnett's multiple comparisons test with adjusted *p* values reported. In comparison to Hep3B cells exposed to normoxia, only *Epo* gene induction values for cells transfected with PPN HIF-2α differed significantly (*p* < 0.0001). In comparison to Hep3B cells exposed to hypoxia, *Epo* gene induction were significantly less for cells transfected with Egr1, Egr2 var2 (Egr2.2), or PPN HIF-1α alone or with either Egr1 or Egr2.2 (*p* < 0.0001). *Epo* gene induction for cells transfected with PPN HIF-2α alone, although elevated, was still significantly less (*p* = 0.0171). However, there was no significant difference (nsd) for *Epo* induction in cells transfected with HIF-2α with Egr1 or Egr2.2. Coexpression of HIF-2α and Egr2.2 approximated hypoxia-induced *Epo* mRNA levels. ∗∗*p* ≤ 0.01. *B*, comparison of *Epo* gene induction by hypoxia in Hep3B cells *versus* control, HIF-1α, HIF-2α, Egr1, or Egr2 knockdown. Data shown are the mean and standard deviation of triplicate rtPCR reactions performed from two wells for each stable cell line. Comparison was assessed by the Brown–Forsythe and Welch's ANOVA tests with Dunnett's T3 multiple comparisons test to adjust *p* values. Reduced *Epo* gene expression was noted following HIF-1α (*p* < 0.0001), HIF-2α (*p* < 0.0001), Egr1 (*p* = 0.0065), and Egr2 (*p* < 0.0001) knockdown. ∗∗*p* ≤ 0.01. *C*, knockdown efficiency assessed by immunoblot. Note that levels of Egr2, a target of Egr1, is reduced by Egr1 knockdown. Egr, early growth response; Epo, erythropoietin; HIF, hypoxia inducible factor.

We performed a series of experiments with Hep3B cells (Fig. 6), a hepatocarcinoma derived cell line that exhibits hypoxia induction of endogenous *Epo* gene expression (54) in a HIF-2 preferential manner (37). We first assayed endogenous *Epo* gene expression in Hep3B cells under normoxia after overexpression of PPN HIF-1α or PPN HIF-2α with WT Egr1 or Egr2 (Fig. 6*A*). Compared to untransfected Hep3B cells maintained under normoxia, *Epo* gene expression increased significantly for all cells transfected with PPN HIF-2α (*p* < 0.0001). Compared to untransfected Hep3B cells exposed to hypoxia, *Epo* gene expression was significantly less than hypoxia for Hep3B cells transfected with Egr1 alone, Egr2 alone, or PPN HIF-1α without or with either Egr1 or Egr2. Although cells transfected with PPN HIF-2α exhibited an increase in endogenous *Epo* gene expression, this value was significantly less than hypoxia treatment (*p* = 0.0171). However, cells transfected with PPN HIF-2α and either Egr1 or Egr2 had augmented *Epo* mRNA levels in Hep3B cells that did not differ significantly from *Epo* induction following hypoxia exposure.

We analyzed the contribution of endogenous HIF-1α, HIF-2α, Egr1, or Egr2 to *Epo* induction during hypoxia following knockdown using short hairpin RNA (shRNA) (Fig. 6*B*). We created stable Hep3B cell lines using lentiviral (LTV) vectors expressing a control shRNA or shRNA targeting HIF-1α, HIF-2α, Egr1 or Egr2. After 8 h of hypoxia exposure and compared to control knockdown Hep3B cells exposed to hypoxia, *Epo* gene expression was reduced with HIF-1α (*p* < 0.0001), HIF-2α (*p* < 0.0001), Egr1 (*p* = 0.0065), or Egr2 (*p* < 0.0001) knockdown. Reduced levels of the targeted protein were evident in each case (Fig. 6*C*). Of note, following Egr1 knockdown, Egr2 levels were also markedly lower, consistent with Egr2 being an Egr1 target gene (41).

**Figure 7:**
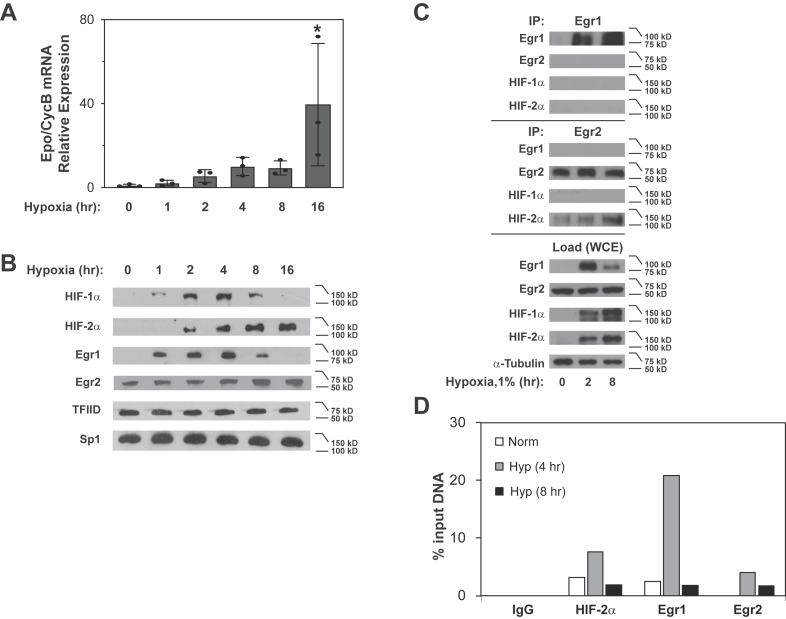
**HIF-1, HIF-2, Egr1, and Egr2 interactions in hypoxic Hep3B cells.***A*, relative *Epo* mRNA levels for a single 6 cm plate of Hep3B cells from each time point measured by rtPCR following a 16 h hypoxia (1% O_2_) time course. Data shown are the mean and standard deviation of three technical replicates performed for each time point. Increased *Epo* gene expression is noted at the 16 h *versus* 0 h time points as assessed by one-tailed unpaired *t* test (*p* = 0.0420). ∗*p* ≤ 0.05. *B*, immunoblots of HIF-1α, HIF-2α, Egr1, and Egr2 in Hep3B cells subjected to a 16 h hypoxia (1% O_2_) time course. *C*, co-immunoprecipitation (co-IP) of endogenous Egr1 and Egr2 with HIF-1α or HIF-2α using extracts prepared from Hep3B cells exposed to 0, 2, or 8 h hypoxia (1% O_2_). *D*, binding of endogenous HIF-2α, Egr1, or Egr2 to the *Epo* enhancer region assayed by chromatin immunoprecipitation (ChIP) for Hep3B cells from 15 cm plates maintained under normoxia (21% O_2_) or hypoxia (4 and 8 h, 1% O_2_). Data shown are the mean of duplicate quantitative PCR measurements of each sample. Egr, early growth response; Epo, erythropoietin; HIF, hypoxia inducible factor.

We defined temporal aspects and physical interactions of Egr1 and Egr2 with HIF-1α and HIF-2α (Fig. 7). We sampled Hep3B cells at various times following hypoxia exposure to confirm *Epo* mRNA induction (Fig. 7*A*) as well as to profile endogenous HIF-1α, HIF-2α, Egr1, and Egr2 proteins (Fig. 7*B*). HIF-1α and Egr1 protein expression peaked early during continuous hypoxia exposure, which was followed later by delayed and sustained induction of HIF-2α and Egr2. We next performed co-immunoprecipitation experiments in Hep3B cells (Fig. 7*C*). During hypoxia Egr2, but not Egr1, formed stable complexes with HIF-2α, but not HIF-1α. HIF-2α, Egr1, and Egr2 were recruited to the *Epo* enhancer during hypoxia as assessed by chromatin immunoprecipitation (ChIP) assays with Egr1 or Egr2 binding to the *Epo* enhancer region enriched after 4 h of hypoxia, like HIF-2α (Fig. 7*D*).

**Figure 8:**
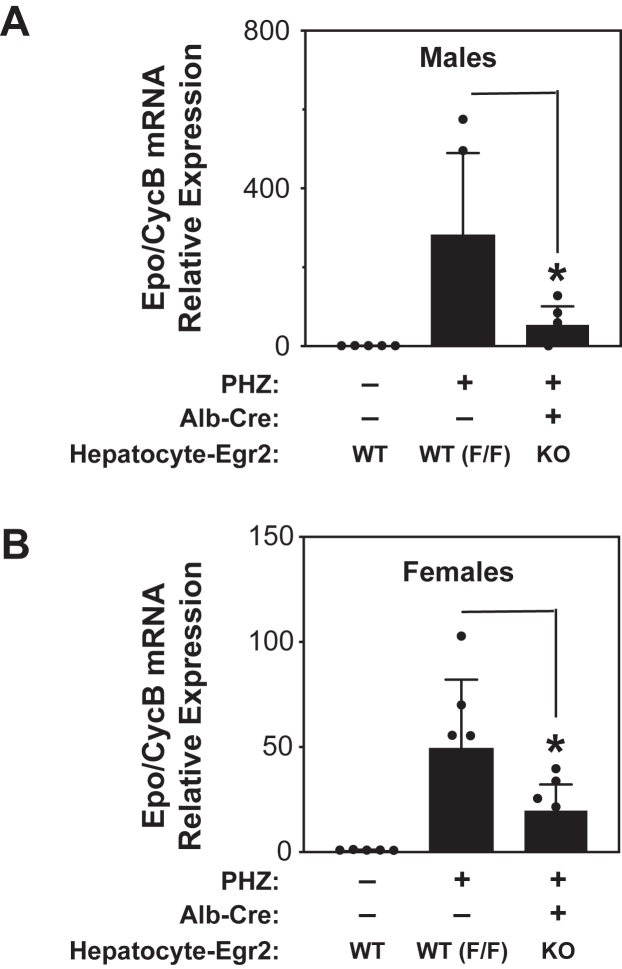
**Egr2 regulates hepatic Epo induction in mice**. Hepatic *Epo* mRNA levels in (*A*) male or (*B*) female mice with or without hepatocyte-specific Egr2 ablation. Floxed *Egr2* mice were crossed with Alb-Cre mice to generate liver-specific *Egr2* KO animals (Alb-Cre+/floxed Egr2 mice). Acute anemia was induced by phenylhydrazine (PHZ) injections in these mice as well as control floxed *Egr2* mice. After 4 days, livers were harvested. Hematocrits were matched between Alb-Cre positive and negative mice and mice chosen with absolute hct<24, which elicits a measurable increase in hepatic *Epo* gene expression in male and female mice. Untreated male WT mice with the same genetic background were used for baseline Epo levels. Data shown are the mean and standard deviation for each genotype. Comparison was assessed by Mann–Whitney test with one-tailed *p* values reported. Alb-Cre positive compared to Alb-Cre negative mice exhibited a significant reduction in hepatic *Epo* mRNA levels for both anemic male (*p* = 0.013) and female (*p* = 0.0469) mice. ∗*p* ≤ 0.05. Egr, early growth response; Epo, erythropoietin.

We hypothesized that Egr2 augments *Epo* gene expression *in vivo*. Phenylhydrazine (PHZ) injections in mice cause an acute hemolytic anemia and induce *Epo* expression. Similar to global *HIF-2*α^*−/−*^ mice (31), *Egr2*^*−/−*^ mice expire during embryogenesis (55, 56). We therefore generated liver-specific *Egr2*^*−/−*^ mice by crossing floxed Egr2 (44) with Alb-Cre mice (57) and measured hepatic *Epo* mRNA levels in anemic mice (Fig. 8). We analyzed male and female mice separately as female mice require higher PHZ doses to induce severe anemia (hematocrit<24) and exhibit lower *Epo* induction levels with a similar degree of anemia. Anemic control (floxed) male mice had significantly elevated *Epo* levels relative to liver-specific *Egr2*^*−/−*^ mice (*p* < 0.0130) (Fig. 8*A*). Similar results were obtained with anemic female liver-specific *Egr2*^*−/−*^ mice (*p* < 0.0469), albeit with lower absolute relative Epo levels (Fig. 8*B*). In summary, these data support Egr2 as a synergistic and directly interacting regulator that acts in conjunction with HIF-2α during hypoxia to increase *Epo* gene expression in Hep3B cells and in mouse liver.

## Discussion

Defining the hypoxia responsive region in the 3′ *Epo* enhancer was a milestone achievement in hypoxia biology. Subsequent studies with transgenic mice indicated that this region regulates late embryonic and neonatal hepatic Epo production (58). HIF-1α and HIF-2α are both present in embryonic liver. Although in mice HIF-1α regulates primitive erythropoiesis (22), hepatic Epo production in late embryonic, juvenile, and adult mice requires HIF-2α (33, 35, 59), which binds an HRE located in the *Epo* 3′ enhancer (58, 60, 61). Although employing a different enhancer (62, 63), renal Epo production in juveniles and adult mice also requires HIF-2α (33, 35), which bind intact HREs located in the *Epo* 5′ upstream region (61, 63). Despite gains in understanding how HIF proteins are regulated and the growing list of essential biological processes influenced by these factors, the molecular mechanisms that dictate HIF-selective signaling remain largely unknown.

To identify a molecular basis for preferential HIF-2 activation of *Epo* gene expression, we first performed homology alignment of 3′ *Epo* enhancers, followed by functional assessments. We identified several conserved sequences, Boxes 1 to 3, containing elements previously implicated in hypoxic or developmental regulation of *Epo* as well as smaller conserved elements located upstream, BSs 1 through 3. Deletion of Boxes 2 and 3 results in increased activation by ectopic oxygen-insensitive HIFs. A primary role of Box 2 is likely regulation of fetal hepatic *Epo* gene expression during mouse embryonic day 9.5 (E9.5) to E12.5 by retinoic acid (64), followed later by hypoxic control (65), which occurs in a HIF-2α rather than HIF-1α dependent manner (59). An inhibitory effect of Box 3 was also evident in studies using integrated marked *Epo* gene cassettes (66) performed in Hep3B cells (11).

We could not demonstrate preferential activation by ectopic HIF-2α of a minimal 3′ *Epo* enhancer, an expanded enhancer containing all highly conserved sequences, or deletion enhancer constructs between these two extremes. We reasoned that the BS123 *cis*-elements recruit additional *trans*-acting factors to confer preferential activation by HIF-2α. Earlier reports described an anemia-induced nuclear factor binding to the BS23 region (67), which showed decreased binding when GC-rich sequences in the 5′ or 3′ end were deleted or replaced. BS1 and BS2 contain potential high and low BSs, respectively, for E-twenty-six (Ets) transcription factor family members that includes several reported to augment signaling by HIF-2, Ets1 (68, 69, 70, 71) and Elk1 (72, 73, 74). A role for Elk1 in *Epo* regulation is supported by knockdown studies in Hep3B cells (73). However, direct binding to the 3′ *Epo* enhancer has not been reported for either Ets1 or Elk1.

BS2 and part of BS3 are also potential BSs for early growth response (Egr) transcription factors. Prior cell studies support a role for Egr in the hypoxic response. Egr1 is involved in growth factor- and hypoxia-regulated responses (41, 43, 75, 76). Egr1 mRNA levels and DNA binding increase during hypoxia in HepG2 cells (77). Egr2 mRNA and protein are upregulated during hypoxia in AC16 cells, a human cardiomyocyte cell line (78). In our study, Egr1 did not transactivate the 3′ *Epo* enhancer reporter in conjunction with PPN HIF-1α or HIF-2α. However, as discussed below, Egr1 may nevertheless impact *Epo* regulation indirectly.

Animal studies also highlight possible links of Egr with Epo regulation. Egr1 mRNA levels increase in embryonic and early postnatal liver at times correlating with hepatic *Epo* expression (79) and is upregulated in the kidney in response to hypoxia or ischemia (80, 81). In addition to human hepatoma cells lines, Egr2 is expressed in rat liver (82). Egr2 is required for normal nervous system development (56) and is expressed in neural crest-derived cell lineages (83), the latter a source of *Epo* producing cells supporting primitive erythropoiesis (84, 85) as well as of *Epo* producing renal interstitial cells involved in definitive erythropoiesis in adult mice (86). Although more restricted in expression compared to Egr2 in mice, Egr3 is required for sympathetic nervous system development (87) and is also expressed in the neural crest like Egr2 (83) and HIF-2 (88).

Egr2 (89) and Egr3 (90, 91) are alternatively spliced in mammals, which results in multiple protein isoforms. We found that Egr2 and Egr3 isoforms differ in their ability to transactivate the 3′ *Epo* enhancer reporter in conjunction with HIF-2α. The shorter Egr2 and Egr3 isoforms maximally stimulated the *Epo* enhancer reporter with HIF-2α although this is not the case with other reporters (91). Multiple isoforms for transcriptional regulators, which typically contain more intrinsically disordered regions (92), may differ in their signaling properties. Indeed, the amino termini of Egr2 and Egr3 are intrinsically disordered and the isoforms examined herein differ in amino terminal length. We could not identify any potential interaction of Egr2 or Egr3 with HIF-2α using artificial intelligence-based protein models such as AlphaFold2. Nevertheless, discerning the functional properties of the intrinsically disordered region in Egr and its potential interaction with HIF-2α is an important topic for future studies of Egr/HIF signaling.

The role of specific isoforms in transcription factors and coactivators is an underappreciated and largely undefined element in most reported gene regulation studies. Although recent systems level studies identified functional differences between transcription factor isoforms (92), a host of factors may complicate applications of conclusions from multiomics studies to regulation of specific genes. For example, if the largest Egr2 and Egr3 isoforms are more stable than the smaller but more transcriptionally active isoforms (with respect to Epo regulation), assessments based on quantitative protein determinations may be misleading. Moreover, conclusions from studies of one regulatory region may not be generalizable to other regulatory regions coregulated by the same two transcription factors for several reasons. While subject to intrinsic and extrinsic determinants including developmental stage, cell type, epigenetic status, and microenvironment, locus-specific responsiveness is largely influenced by the specific architecture of associated regulatory regions and the related recruitment of a unique set of genetic regulatory factors, which may include specific transcription factor isoforms.

We propose that Egr1 and select isoforms of Egr2, and possibly Egr3, act with HIF-2 to regulate *Epo* gene expression during hypoxia. Egr1 and HIF-1α protein levels exhibited a coordinated increase in Hep3B cells exposed to hypoxia. Egr2 and HIF-2α likewise increased in a similar fashion, albeit in a delayed manner that paralleled the rise in *Epo* mRNA expression. Knockdown of endogenous Egr2, and to a lesser degree Egr1, blunted *Epo* induction during hypoxia in Hep3B cells. Ectopic expression of HIF-2α with Egr1 or Egr2 in Hep3B cells induced *Epo* to levels that rivaled those observed following hypoxia exposure. Egr2 interacts directly with HIF-2α whereas Egr1 acts indirectly *via* induction of Egr2 during hypoxia. Egr1 and Egr2 are both recruited to the 3′ *Epo* enhancer in Hep3B cells exposed to hypoxia. Although synergy between Egr1 and endogenous HIF-1α may be relevant in the early hypoxic phase when both are expressed (76, 77), Egr2 clearly plays an *in vivo* role in *Epo* gene regulation as Egr2 ablation in liver resulted in blunted hepatic *Epo* gene expression in anemic mice.

As evident in our knockdown studies in Hep3B cells, Egr1 is required for maintenance and induction of Egr2 in cells exposed to hypoxia. Other cellular factors may also modulate Egr1 and Egr2 levels. Egr1 and Egr2 expression in mouse embryonic stem cells are controlled by Elk1 and the associated ternary complex (93). Knockdown of Elk1 impairs Egr1 in vascular smooth muscle cells (94) or Egr2 in mouse embryonic fibroblasts as well as in 3T3-L1 preadipocytes (95). Thus, by regulating expression of Egr1 or Egr2, Elk1 knockdown (72) may indirectly impact *Epo* induction by HIF-2 in Hep3B cells.

One mystery regarding *Epo* regulation in mammals is how it is rapidly turned on and off. Although *Epo* gene expression initially and rapidly increases following a hypoxic insult, serum Epo protein and renal *Epo* mRNA levels decrease rapidly despite continued hypoxia exposure and within hours after the onset of hypoxia, well before a change in red blood cell mass occurs and despite stabilization of HIF members (96, 97). Epo induction may be regulated in part by autocrine signaling. Hep3B cells treated with recombinant human Epo induced endogenous *Epo* expression under normoxic conditions (98). Furthermore, Epo upregulates Egr1 (99) and Egr2 (100) in cell studies through Epo receptor signaling. Thus, if *Epo* gene expression is initially induced by HIF transactivation, Epo protein may then stimulate expression of Egr factors in an autocrine manner, further augmenting *Epo* induction *via* Egr/HIF-2 transactivation.

*Epo* induction may also be affected by multiple epigenetic and genetic mechanisms. Although methylation of the 3′ *Epo* enhancer is associated with a quiescent state (101, 102), Egr1 recruitment is not affected by CpG methylation (103, 104) and Egr2 initiates DNA demethylation at its BSs (105). Moreover, Egr1 and Egr2 (49, 106) also recruit cellular factors that modify *Epo* chromatin to alter its accessibility. In contrast, Egr1 and Egr2 also interact with NAB1 (47) and NAB2 (52) proteins, themselves downstream targets of Egr signaling (53, 107, 108, 109), to recruit epigenetic modulators (110, 111, 112, 113) similar to HIFs (114) that may silence chromatin. With respect to Egr2 and HIF-2 themselves, Egr2 is acetylated and deacetylated by Cbp and Sirt1, respectively (115), like HIF-2α (59, 116), which modulate their transactivation capacity. Thus, if downstream regulators of Egr signaling also modulate HIF-2 signaling, Egr/HIF-2 signaling may provide a molecular platform for not only rapidly increasing, but also for preventing physiological overshoot of Epo induction and a resultant erythrocytosis with associated thrombotic events.

## Experimental procedures

### Bioinformatics

Sequence for the Epo enhancer was obtained using genome browsers available from NCBI (http://www.ncbi.nlm.nih.gov/) and UCSC (http://genome.ucsc.edu). Genome alignment analysis was performed using rVista (37). The enhancer region was visually analyzed using a ClustalW multiple sequence alignment program (http://www.ebi.ac.uk/cgi-bin/clustalw). Putative BSs in conserved regions were identified with the Tfsitescan/dynamic Plus server (39).

### Reporter and expression plasmids

The mouse *Epo* enhancer/promoter reporters were generated by PCR using mouse genomic DNA or precursor plasmids. The mouse *Epo* enhancer and promoter segments were cloned into pGL3-Basic (Cat. No. E1751, Promega) *via* a three-way ligation using NheI/XhoI enhancer fragments and SalI/HindIII promoter fragments. Mutated bases are indicated in small text and underlined with a dotted line. The WT mouse *Epo* (−410/+1) promoter sequence amplified from mouse genomic DNA contains Sal I and Hind III sites (small text) at the 5′ and 3′ ends, respectively: gtcgacTGCCTGGAACAGCCTGCTCCACCCCAGCAAGCACCAGACCCAGGCGTCCTGCCCCTTGCTCTGACCCCAGGTGGCCCCCACCTCTGGCGACCCCTCACGCACACACAGCTTCACCCCCACCCCCACCCGCGCACGCACACATGCTGATAACATCCCCGACCCCCGGCCGGAGCCACAGTGTCCCGGGACCAACCCTGGCCGGTGGCTGTGTCTCACTGTGTTCCCGAACGGACCCTTGGCCAGGGCCACCGCGTCCCCACTCTGCCCGCGCCCCCTGGACAGTGACCACTTTCTTCCAGGCTAGTGGGGTGATCTGGCCCTACAGAACTTCCAAGGATGAAGACTTGCAGCGTGGACACTGGCCCAGCCCCGGGTCGCTAAGGAGCTCCGGCAGCTAGGCGCGGAGaagctt. The WT mouse *Epo* enhancer in BS123-Box123 mEpoEnh-mEpoProm-luc (pp1176 and pp4376) amplified from mouse genomic DNA contains an Nhe I and Xho I site at the 5′ and 3′ ends, respectively, with the HRE in bold, underlined text. The (BSs 1 to 3 proceed from 5′ to 3′ and are indicated by double underlined, italicized text with BS3 encompassing the HRE: gctagcAGATCT*GGGAAACCAG*A*GGTGGAGGGGG*T*TGGGCCCT****ACGTG****C*TGCCTCGCATGGCCCGGCTGACCTCTTGACCCCTCTGGGCTTGAGGCCACAATGCCTGCCCACGCTAGTCAATAAGCAGGCTCCATTCAAGGCTGTCTCTCAGTGGGCAGCTGCCAACCCTcgag. The WT mouse *Epo* enhancer in BS123-Box12 mEpoEnh-mEpoProm-luc (pp1177) was generated by PCR using mouse genomic DNA and in BS123-Box1 mEpoEnh-mEpoProm-luc (pp1178) was generated by PCR from BS23-Box1 mEpoEnh-mEpoProm-luc (pp901). The WT mouse *Epo* enhancer in BS23-Box1 mEpoEnh-mEpoProm-luc (pp901) was amplified from pp1176 with an Nhe I and an Xho I site at the 5′ and 3′ ends, respectively: gctagc*AAACCAG*A*GGTGGAGGGGG*T*TGGGCCCT****ACGTG****C*TGCCTCGCATGGCCCGGCTGACCTCTTGACCCCTCgag. The WT mouse *Epo* enhancer in BS3-Box1 mEpoEnh-mEpoProm-luc (pp992) was generated by PCR from BS23-Box1 mEpoEnh-mEpoProm-luc (pp901). The WT mouse Epo BS123-Box123 enhancer reporter used in the HIF-Egr cotransfection experiments (pp4451) is identical to the BS23-Box1 mEpoEnh-mEpoProm-luc reporter (pp901) except that the junction with the downstream mouse promoter is *via* an Xho I site. The mutant BS23 mouse Epo enhancer sequence in mBS23 BS123-Box123 (pp4452) was generated from this construct by site-directed mutagenesis and contains the following sequence: gctagc*AAACCAG*A**cta**G**a**A**ttcct**T*TGGGCCCT****ACGTG****C*TGCCTCGCATGGCCCGGCTGACCTCTTGACCCCTCgag. Expression plasmids encoding mutant oxygen-independent (PPN) HIF-1α and HIF-2α, were previously described (95). Egr1 (NP_031939.1), Egr2 isoform 1 (Egr2.1) (NP_034248.2) and isoform 2 (Egr2.2) (NP_001334387.1), Egr3 isoform 1 (Egr3.1) (NP_061251.1) and isoform 3 (Egr3.2 in this study) (NP_001276856.1), and Egr4 (AK141637) encoding expression plasmids were generated from mouse Egr1 (J04089), Egr2 (AK153882), Egr3 (BC103568), and Egr4 (AK141637) EST plasmids with use of extension PCR in some cases. Isoforms 1 for mEgr2 and mEgr3 share extensive homology as do isoforms 2 and 3 for mEgr2 and mEgr3, respectively, with the latter isoforms encoding amino-truncated versions of isoform 1 for both Egr2 and Egr3. PPN HIF expression constructs have a carboxy terminal HA epitope tag. The tagged PPN HIF proteins retain intact transactivation properties compared to the untagged forms as judged by transient transfection assays. Mutations were introduced using PCR-based site-directed mutagenesis (QuikChange II XL; Cat. No. 200521, Agilent) and sequence-verified prior to cloning. All expression plasmids, unless otherwise indicated, were cloned into pIRES-hrGFP-2a (Cat. No. 240032, Stratagene).

### Lentivirus

The LTV knockdown constructs express multiple miR-30 shRNAs for efficient knockdown (96, 97). For knockdown studies in Hep3B cells, in LTV shuttle expression vectors (derivatives of pLenti6/V5-GW/lacZ, Cat. No. V49610, Invitrogen), the blasticidin resistance gene was replaced with a neomycin resistance gene, and DsRed was inserted followed by a 3′ UTR containing a concatemer of four different shRNAs directed against the gene of interest. The seed sequences for the two control shRNAs arranged in tandem for a total of four inserts are as follows: 5′- TACATCCCGATCGATGATG-3′, 5′- TCGCTTGGGCGAGAGTAAG-3′. The seed sequences for the four human HIF-1α shRNAs are as follows: 5′-GAACAAATACATGGGATTA-3′, 5′-AGAATGAAGTGTACCCTAA-3′, 5′-GATGGAAGCACTAGACAAA-3′, 5′-CAAGTAGCCTCTTTGACAA-3′ The seed sequences for the four human HIF-2α are as follows: 5′-ACTGCTATCAAAGATGCTGTTC-3′, 5′-TCTGTGTCCATGGCGAAGAGCT-3′, 5′-TTCATACTCCAGCTGTCGCTTC-3′, 5′-TAAGTCTATCCGGGCTTACTAA-3′ with this last shRNA targeting the 3′ untranslated region of the mRNA. The seed sequences for the four human Egr1 shRNAs are as follows: 5′-GATGAACGCAAGAGGCATA-3′, 5′-CGACAGCAGTCCCATTTAC-3′, 5′-GGACATGACAGTAACCTTT-3′, 5′-GACCTGAAGGCCCTCAATA-3′. The seed sequences for the four human Egr2 shRNAs are as follows: 5′-GAAGGCATAATCAATATTG-3′, 5′-CTACTGTGGCCGAAAGTTT-3′, 5′-GAAACCAGACCTTCACTTA-3′, 5′-GAGAAGAGGTCGTTGGATC-3′. The LTV packaging plasmid psPAX2 (Cat. No. 12260, Addgene) and the envelope plasmid pMD2.G (Cat. No. 12259, Addgene) were used in conjunction with the shuttle expression plasmids to generate lentivirus. Subsequently, 10-cm plates of HEK293T cells (American Type Culture Collection, ATCC, Cat. No. CRL-3216) were cotransfected with 3 μg LTV shuttle expression vector, 2.25 μg psPAX2 and 0.75 μg pMD2G per plate using polyethylenimine (PEI; Cat. No. 23966, Polysciences) at a ratio of 3.5 μl/μg DNA. Virus was concentrated from cell media collected over 3 days using Lenti-X Concentrator (Cat. No. 631232, Clontech). The virus was aliquoted after gentle resuspension in culture media and stored at −80 °C until use.

### Stable cell lines

To generate stable cell lines, Hep3B cells were plated in 12-well plates at 2 × 10^5^ cells per well the day before transduction. On the day of transduction, complete medium with polybrene (Cat. No. 107689, Sigma-Aldrich) was prepared at a final polybrene concentration of 10 μg/ml. Media were removed from the wells and replaced with 1 ml of the polybrene/media mixture. Lentiviral particles were added to the Hep3B cells at an estimated multiplicity of infection = 30 and then incubated overnight. The following day, the culture medium was replaced with 1 ml of complete medium containing 400 μg/ml G418 (Cat. No. SV30069, HyClone) and replaced every 2 days until 1 week after all control cells died, 2 weeks total. After initial selection, cells were maintained at 100 μg/ml G418, which was omitted when cells were plated for experiments.

### Cell culture

HEK293T (ATCC, Cat. No. CRL-1573) and Hep3B cells (Cat. No. HB-8064, ATCC) were maintained in complete media [Dulbecco's modified Eagle's medium, 4.5 g/l glucose, 4 mM glutamate (Cat. No. SH30022, HyClone), 10% fetal bovine serum (Cat. No. S10650H, Atlanta Biologicals) supplemented with penicillin (100U/ml)/streptomycin (100 μg/ml) (Cat. No. 15140-148, Gibco BRL)] in a 5% CO_2_, 95% air incubator. For hypoxia treatments, cells were transferred to a humidified environmental chamber (Coy Laboratory Products, Inc), culture media replaced with deoxygenated media, and cells maintained under hypoxic conditions (1% O_2_, 5% CO_2_, and 94% N_2_) for the specified periods. Cells were harvested within the hypoxia chamber. Cells are tested periodically for *mycoplasma* and were free from *mycoplasma* during these experiments. Cells were purchased from ATCC and are periodically assessed to confirm their identity by genetic analyses.

### Luciferase reporter assays

For luciferase reporter experiments, HEK293T cells were plated on 48-well plates at 100,000 cells/well or 96-well plates at 40,000 cells/well. After 24 h, cells were transfected in triplicate with 330 ng total DNA (30 ng reporter + 100 ng each expression plasmid) per well on 48-well plates or 167 ng total DNA per well on 96-well plates (17 ng reporter + 50 ng each expression plasmid) using Lipofectamine 2000 (Cat. No. 11668–019, Invitrogen) with the balance of DNA made by empty expression vector (pIRES-hrGFP-2a). At 24 h post transfection, cells were harvested for luciferase assays. For 48-well plates, at harvest, media were aspirated from cells and 50 μl lysis buffer was added, followed by 30 min incubation at room temperature. Lysate was then mixed with substrate buffer at a 1:5 ratio and immediately read on a CLARIOstar Plus plate reader (BMG Labtech). Core lysis buffer was prepared in molecular grade water with 30 mM tricine (pH 7.8), 8 mM magnesium acetate, 0.2 mM EDTA, 1% Triton X-100, and 100 mM 2-mercaptoethanol. Substrate buffer was prepared by supplementing lysis buffer with 2.5 mM magnesium chloride, 1.5 mM ATP, 0.5 mM coenzyme A, and 0.5 mM D-Luciferin. For 96-well plates, One-Glo reagent (Cat. No. E6110, Promega) was added directly to the plate at a 1:1 ratio of reagent to media. Cells were incubated at room temperature for 5 min before reading on a CLARIOstar Plus plate reader. Results are an average of three separate experiments performed on different days.

### Immunoblot analyses

To prepare whole cell protein extracts, cells were first rinsed with PBS, and then lysed with CytoBuster protein extraction reagent (Cat. No. 71009, Novagen) supplemented with 1× protease inhibitor cocktail (Cat. No. P8340, Sigma-Aldrich). Cells were scraped into a microfuge tube, cell debris pelleted, and protein-containing supernatant transferred to a new tube. Protein concentration was determined by BCA protein assay (Cat. No. 23225, Thermo Fisher Scientific). For Western blotting, SDS-PAGE was performed on extracts, followed by electrophoretic transfer to polyvinylidene fluoride. Immunostaining was performed with the following commercially available antibodies: Egr1 (Cat. No. sc-110, Santa Cruz Biotechnology); Egr2 (Cat. No. PRB-236P, Covance); HIF-1α (Cat. No. 610959, BD Biosciences); HIF-2α (Cat. No. NB100-132, Novus Biologicals); α-tubulin (Cat. No. T9026, Sigma-Aldrich); TFIID (Cat. No. sc-204, Santa Cruz Biotechnology); Sp1 (Cat. No. sc-59, Santa Cruz Biotechnology). Validation was initially provided by the manufacturer and was confirmed following knockdown. The following secondary antibodies were used: horseradish peroxidase (HRP)-linked anti-rabbit immunoglobulin G (IgG) (Cat. No. 7074, Cell Signaling Technology), HRP-linked anti-mouse IgG (Cat. No. 7076, Cell Signaling Technology), HRP-linked mouse anti-goat IgG (Cat. No. sc-2354, Santa Cruz Biotechnology). Following secondary antibody binding, membranes were incubated with Clarity (Cat. No. 1705060, Bio-Rad), SuperSignal West Dura (Cat. No. 34076, Thermo Fisher Scientific), or SuperSignal West Femto (Cat. No. 34094, Thermo Fisher Scientific) luminol enhanced chemiluminescence reagents, exposed to X-ray film and developed on an automatic film developer.

### Endogenous co-immunoprecipitation experiments

Endogenous Egr1 or Egr2 were immunoprecipitated using a Universal Magnetic Co-IP kit (Cat. No. 54002, Active Motif). Hep3B cells were incubated under normoxic or hypoxic (1% O_2_, 5% CO_2_, and 94% N_2_) conditions for 2 and 8 h before harvest. Hypoxia-treated samples were harvested within the hypoxia chamber and then removed for pelleting and lysis. Whole-cell extracts (500 μg total protein) were precleared with magnetic protein G beads, then incubated with antibodies to Egr1 (Cat. No. 4153, Cell Signaling Technology) or Egr2 (Cat. No. ab63943, Abcam) for 2 h at 4 °C on a rotator. Magnetic protein G beads were added and incubated for 1 h before washing. The beads were washed and eluted according to kit instructions and immunoblotted with antibodies to HIF-1α (Cat. No. 610959, BD Biosciences) or HIF-2α (Cat. No. NB100–132, Novus Biologicals). Whole cell extract was used as a loading control and immunoblotted with antibodies to HIF-1α, HIF-2α, EGR1, EGR2, and α-tubulin (Cat. No. T9026, Sigma-Aldrich).

### Chromatin immunoprecipitation (ChIP) assays

Hep3B cells seeded at 2 × 10^6^ (150 mm plates) 48 h prior to use were exposed to normoxia or hypoxia for 4 or 8 h, then harvested for whole cell protein or RNA. *Epo* induction was confirmed by real-time RT-PCR. ChIP assays were carried out using the ChIP-IT Express Magnetic assay kit (Cat. No. 53009, Active Motif). The antisera were normal mouse IgG (1–2 μg/ml; Cat. No. sc-2025, Santa Cruz Biotechnology), normal rabbit IgG (1–2 mg/ml; Cat. No. NI01, EMD Chemicals, Inc), anti-human EPAS1/HIF-2α antiserum (2 mg/ml; Cat. No. NB100–132, Novus Biologicals), and rabbit anti-Egr1 (Cat. No. sc-110, Santa Cruz Biotechnology) or anti-Egr2 (Cat. No. sc-20690, Santa Cruz Biotechnology). After ChIP, precipitated genomic DNA was analyzed in triplicate by quantitative PCR with an Applied Biosystems ABI Prism 7000 thermocycler (Applied Biosystems) and Power SYBR Green Master Mix (Cat. No. 4367659, Applied Biosystems) using the following primers for the human *Epo* enhancer: 5′-ACTCCTGGCAGCAGTGCAGC-3′ (forward) and 5′-CCCTCTCCTTGATGACAATCTCAGC-3′ (reverse). Captured genomic DNA was normalized to input material for each antibody and compared between normoxic and hypoxic treatments.

### Real time RT-PCR analyses

Endogenous Epo and cyclophilin expression was determined by reverse transcription of total RNA followed by real-time PCR (rtPCR) analysis. Total RNA from Hep3B cells was extracted using GenElute mammalian Total RNA kit (Cat. No. RTN70-1KT, Sigma-Aldrich) or Aurum Total RNA mini kit (Cat. No. 7326820, Bio-Rad). One microgram of total RNA was reverse transcribed using iScript complementary DNA (cDNA) Synthesis kit (Cat. No. 1708891, Bio-Rad) according to the manufacturer's protocol. Total RNA from the mouse liver or kidney was extracted from 30 mg of tissue using the Aurum Total RNA mini kit. Subsequently, 1 μg of total RNA was then reverse transcribed using iScript cDNA Synthesis kit. Values were normalized to cyclophilin as previously described (95). rtPCR was performed on an Applied Biosystems ABI Prism 7000 thermocycler using SYBR GreenER qPCR SuperMix (Cat. No. 11760, Invitrogen) or PowerUp SYBR Green Master Mix (Cat. No. A25742, Thermo Fisher Scientific) following the manufacturer's protocol and one percent of total cDNA with the following pairs of human primers: Epo (forward) 5′-GAGGCCGAGAATATCACGACGGG-3′, Epo (reverse) 5′-TGCCCGACCTCCATCCTCTTCCAG-3′, cyclophilin (forward) 5′-ATGTGGTGTTTGGCAAAGTTCTA-3′, cyclophilin (reverse) 5′-GGTTTATCCCGGCTGTCT-3′, or mouse primers: Epo (forward) 5′-GAGGCAGAAAATGTCACGATG-3′, Epo (reverse) 5′-CTTCCACCTCCATTCTTTTCC-3′, cyclophilin (forward) 5′-ATGTGGTTTTCGGCAAAGTTCTA-3′, cyclophilin (reverse) 5′-GGCTTGTCCCGGCTGTCT-3′. The results of triplicate experiments were expressed as 2^(-(EPO number of cycles- cyclophilin number of cycles))^.

### Mouse PHZ experiments

To generate mice with Egr2 deleted in the liver, liver-specific Alb-Cre mice (45) (JAX stock #003574, The Jackson Laboratory) were crossed to mice with a floxed Egr2 gene (44). Cre negative offspring were used as WT littermate controls. Acute, hemolytic anemia was induced at 10 to 11 w.o. mice with serial subcutaneous phenylhydrazine (Cat. No. 114715, Sigma-Aldrich) injections administered subcutaneously (intrascapular) to isoflurane-anesthetized mice (2% using a vaporizer). PHZ concentration was adjusted in PBS for an injection volume of 8 μl/g body weight. Two doses were administered spaced 24 h apart. Males received 65 μg PHZ/kg body weight. Females were more resistant to PHZ and thus required a higher initial dose of 80 μg/kg body weight followed by a second dose of 65 μg/kg. Four days after the first PHZ injection, hematocrits were measured in duplicates with blood from retro-orbital eye bleeds. Mice with hematocrits ranging from 17% to 24%, defined as severe anemia, were harvested for further study. All studies were approved by the UTSW Institutional Animal Care and Use Committee.

### Statistical analyses

Statistical analyses were performed using Prism (Graphpad Software). Data were presented as mean with standard deviation (SD) or standard error of the mean (SEM). We used ordinary one-way ANOVA analyses for multiple comparisons of experimental groups with equal variances confirmed by Brown–Forsythe and Bartlett's test. For comparisons to one control, we used Dunnett's multiple comparisons test to adjust *p* values. For comparisons between all groups, we used Tukey's multiple comparisons test to adjust *p* values. For comparisons between preselected samples, we used Sidak's multiple comparisons test to adjust *p* values. We used the Brown–Forsythe and Welch's ANOVA tests for multiple comparisons of experimental groups with unequal variances and Dunnett's T3 multiple comparisons test to adjust *p* values. For pair-wise comparisons, we used the two-tailed Welch's *t* test for cell culture experiments and the one-tailed Mann-Whitney test for mouse experiments. All findings were rounded to four significant digits. *p* values considered significant are less than or equal to 0.05.

## Data availability

Original collated data from all transfections, rtPCR, and ChIP assays, as well as statistical calculations performed are provided in a supplemental Excel file. Images from original immunoblots are available upon request.

## Supporting information

This article contains supporting information.

## Conflict of interest

The authors declare that they have no conflicts of interest with the contents of this article.

## Supporting information

Nagati Dioum_JBC_data_raw_rev2

Nagati Dioum_JBC_Fig6C_IB scans

Nagati Dioum_JBC_Fig7B_IB scans

Nagati Dioum_JBC_Fig7C_IB scans
