# Supplementary material for: Select early growth response (Egr) isoforms augment hypoxia inducible factor 2 (HIF-2) regulation of erythropoietin (Epo) gene expression in mammals[image]
Source: J Biol Chem. 2025 Jun 10;301(7):110355. doi: 10.1016/j.jbc.2025.110355 (PMC12274846; doi:10.1016/j.jbc.2025.110355)
Supplement: Nagati Dioum_JBC_Fig6C_IB scans [file mmc2.pdf]

Hep3B Stable cell lines
Hif, Egr Knockdown IB: Hif-1α

Hep3B stable cell lines
30ug WCE
8 hrs hypoxia

1:1000, ON
1:3000 2°, 1hr

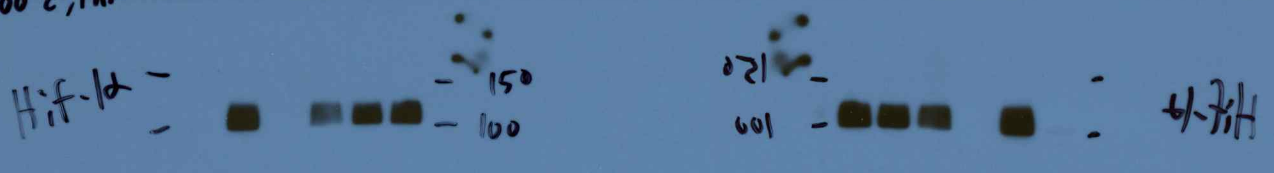

1 2 3 4 5 6

Plasmid	K.D. target	
1. 2648	ctrl	normoxia
2. 2648	ctrl	8hrs hypoxia
3. 2649	Hif-1α	"
4. 2650	Hif-2α	"
5. 2651	Egr1	"
6. 2652	Egr2	"

1:1000 Hif-1α AB
BD Bio #610959

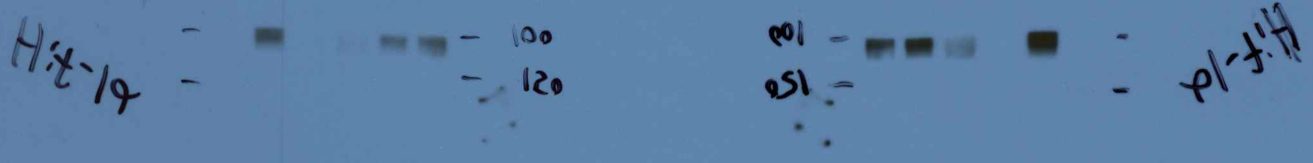

Hep3B SCLs
Hif, Egr K.D. IB: Hif-2α

Hep3B stable cells
WCE, normoxia vs. hypoxia
Knockdown efficiency

Hif-2α 1°AB

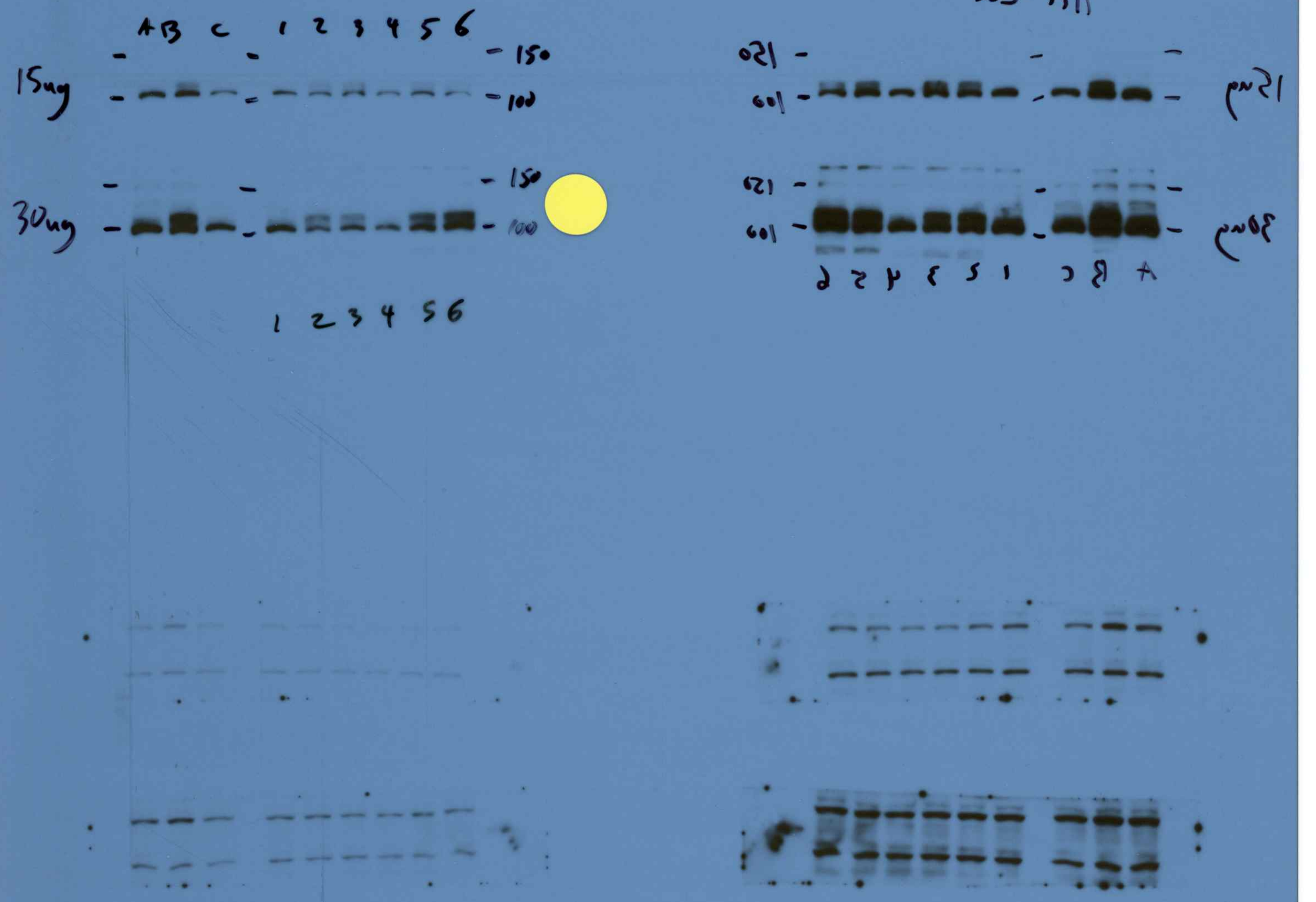

1. 2648 ctrl, normoxia
2. 2648 ctrl, 8hrs hypoxia
3. 2649 Hif-1α, 8hrs hypoxia
4. 2650 Hif-2α, " "
5. 2651 Egr1, " "
6. 2652 Egr2, " "

2 min

Hep3B SCLs
Hif, Egr KD IB: Egr1

Hep3B shRNA SCLs
0,8 hrs hypoxia
30ug WCE

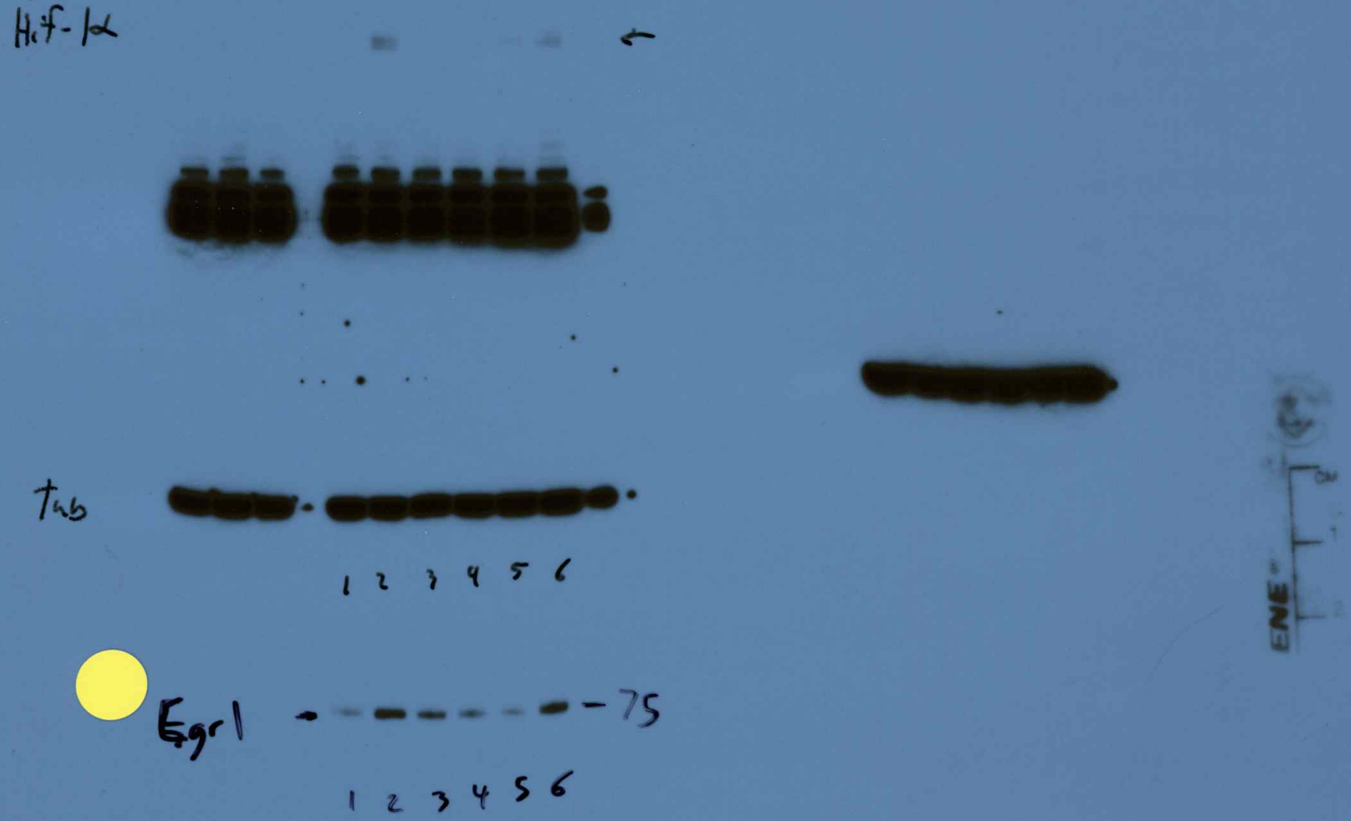

Plasmid (shRNA)		Hypoxia (hrs)
1. 2648	ctrl	0
2. 2648	ctrl	8
3. 2649	Hif-1α	8
4. 2650	Hif-2α	8
5. 2651	Egr1	8
6. 2652	Egr2	8

ABs
Egr1 - 1:1000, SCBT #sc-110
Tub - 1:10,000, Sigma #T9026

Hep3B SCLs w/shRNA
Hif, Egr Knockdown IB: Egr2

Egr2 Antibody
Covance 1:1000
Hep3B WCE

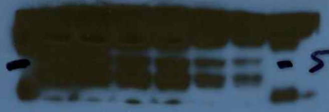

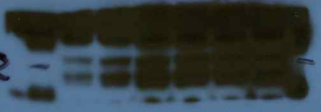

1 2 3 4 5 6

Lane	Knockdown
1.	Ctrl norm
2.	Ctrl hypo
3.	Hif-1a
4.	Hif-2a
5.	Egr1
6.	Egr2

(Lanes 2–6: 8hrs hypoxia)

Hep3B shRNA Stable cell lines
0,8 hrs hypoxia
30ug whole cell extract
Egr2 antibody, 1:1000, Covance #PRB-236P

bottom of membrane

Egr2 →

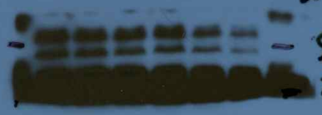

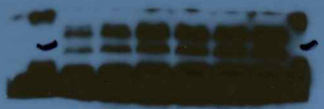

Top of membrane

1 2 3 4 5 6

Hep3B SCLs
Hif, Egr KD IB: tubulin

1 sec

Hep 3B stable cell lines
30ug WCE
8 hrs hypoxia

1:10,000 αTubulin
Sigma, #T9026

Hif-2α

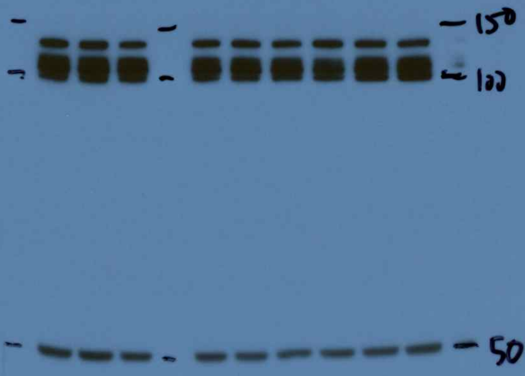

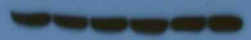

Tub

A B C 1 2 3 4 5 6

1. 2648 ctrl normoxia
2. " " 8 hrs hypoxia
3. 2649 Hif-1α "
4. 2650 Hif-2α "
5. 2651 Egr1 "
6. 2652 Egr2 "

A. No plasmid; 0 hrs hypoxia
B. " 8 hrs "
C. 2734 Hif-2α(A+B+C+D shRNA) 8 hrs hypoxia
