# Supplementary material for: Select early growth response (Egr) isoforms augment hypoxia inducible factor 2 (HIF-2) regulation of erythropoietin (Epo) gene expression in mammals[image]
Source: J Biol Chem. 2025 Jun 10;301(7):110355. doi: 10.1016/j.jbc.2025.110355 (PMC12274846; doi:10.1016/j.jbc.2025.110355)
Supplement: Nagati Dioum_JBC_Fig7B_IB scans [file mmc3.pdf]

Hep3B hypoxia TC
IB: Hif-1α

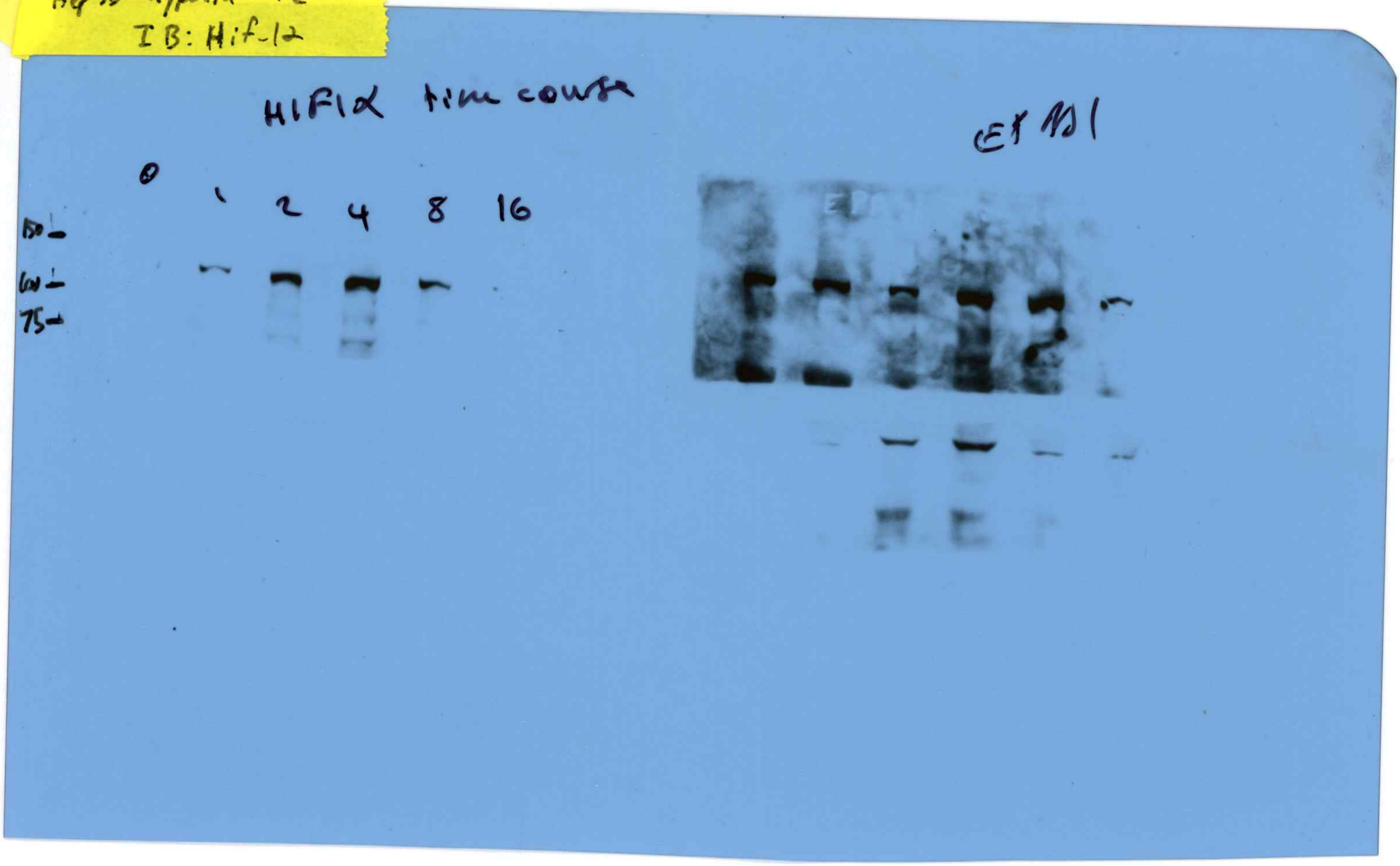

IB: Epas1
(Hif-2α)

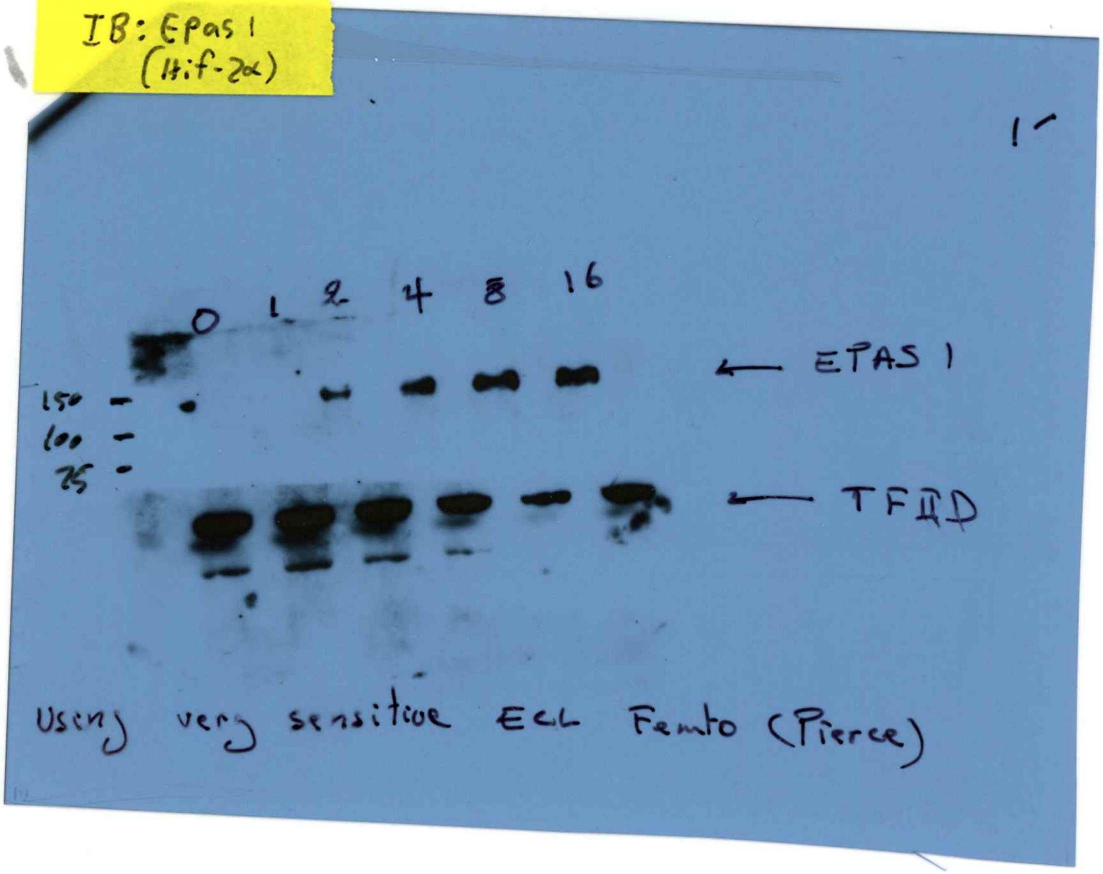

Hep 3B Hypoxia T.C.
IB: Egr1

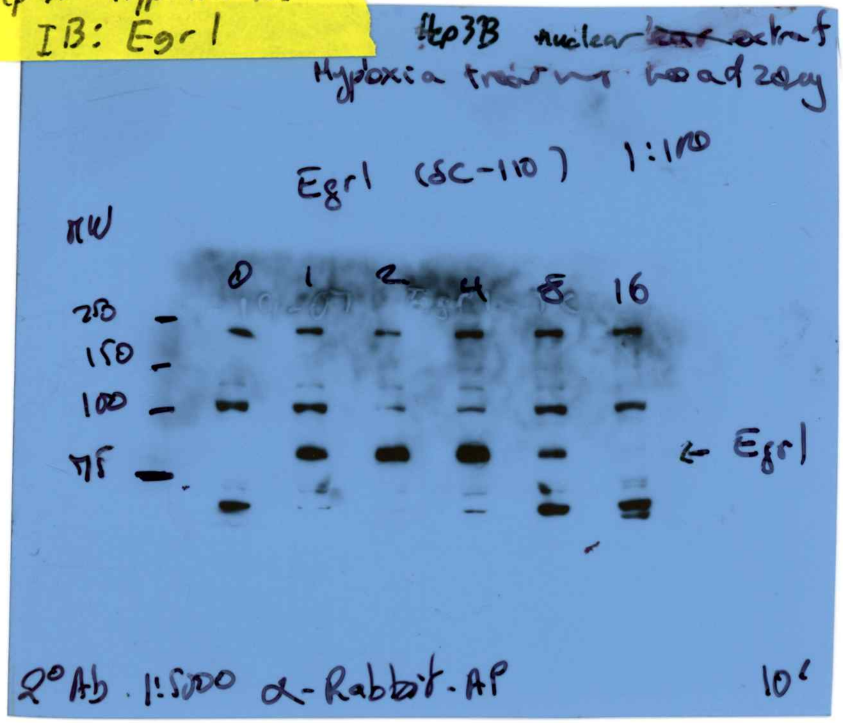

IB: TFIID

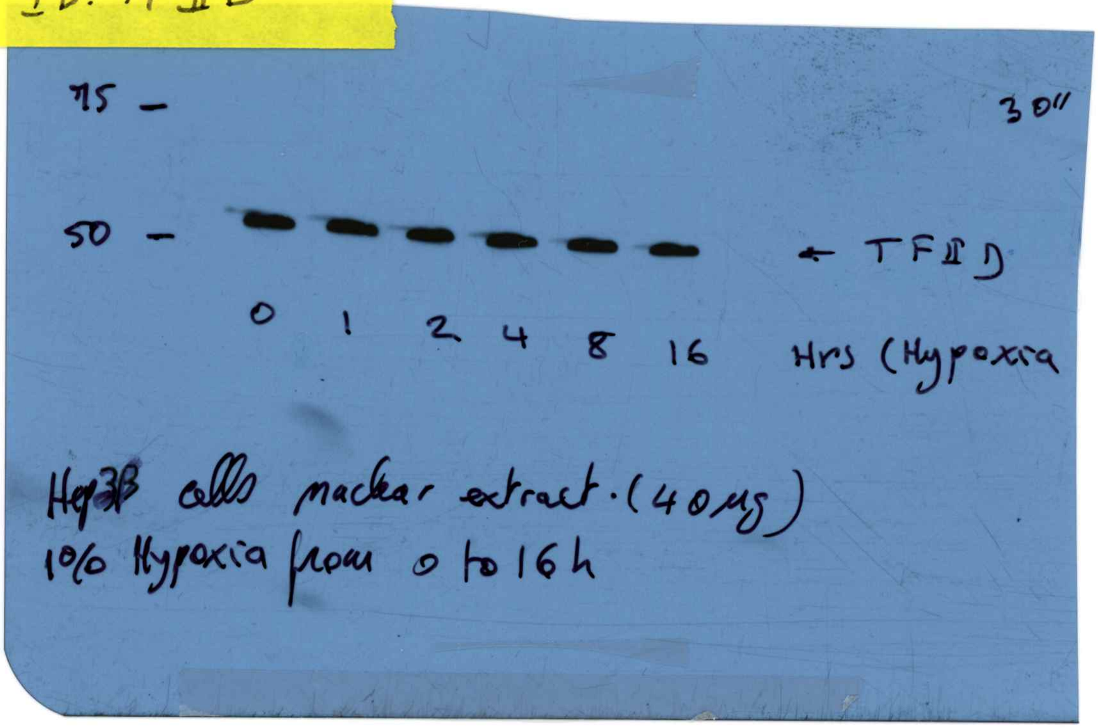

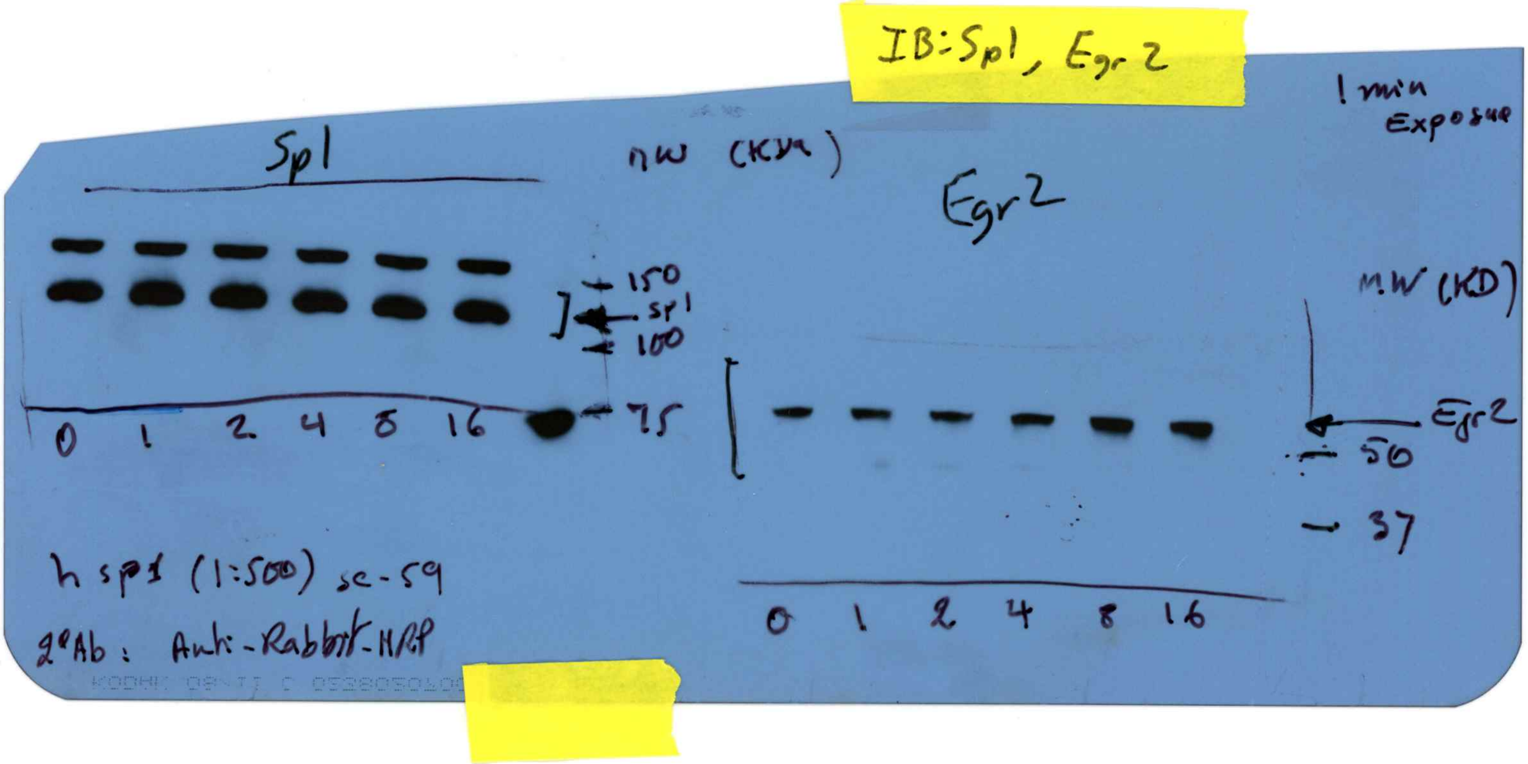
IB: Sp1, Egr2
1 min Exposure
Sp1
MW (KDa)
Egr2
150
Sp1
100
75
0 1 2 4 8 16
MW (KD)
Egr2
50
37
0 1 2 4 8 16
h sp1 (1:500) sc-59
2°Ab: Anti-Rabbit-HRP
