# Supplementary material for: Select early growth response (Egr) isoforms augment hypoxia inducible factor 2 (HIF-2) regulation of erythropoietin (Epo) gene expression in mammals[image]
Source: J Biol Chem. 2025 Jun 10;301(7):110355. doi: 10.1016/j.jbc.2025.110355 (PMC12274846; doi:10.1016/j.jbc.2025.110355)

CoIP - P.D. Egr1, Egr2   Hep3B - 0,2,8 hrs hypoxia
P.D. Egr1, Egr2 , IB: Egr1, Egr2

10sec

IP/IB/Egr2

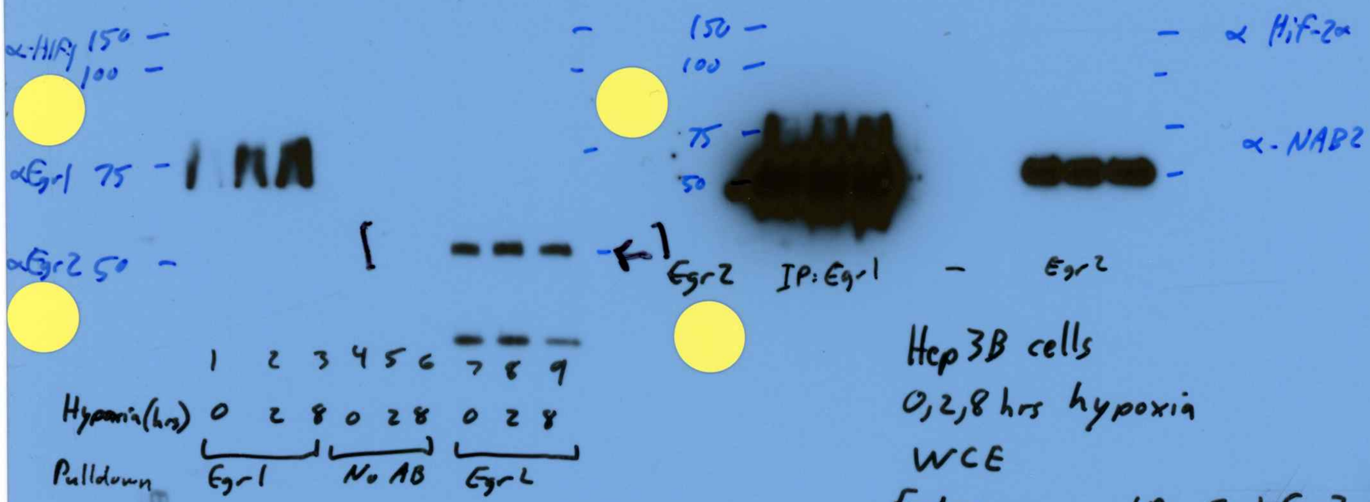

Hep3B cells
0,2,8 hrs hypoxia
WCE
Endogenous co-IP - Egr1, Egr2, No AB
All 1° ABs 1:1000, ON
GAM-HRP (Hifs, NAB2) 1:3000, 1hr RT
GAR-HRP (Egr1) 1:3000, 1hr, RT
MAG-HRP (Egr2) 1:6000, 1hr, RT

WB, IP ABs:
Egr1 - CST # 4153
Egr2 - abcam ab63943
Hif-1α - BD Bio 610959
Hif-2α - Novus NB100-132

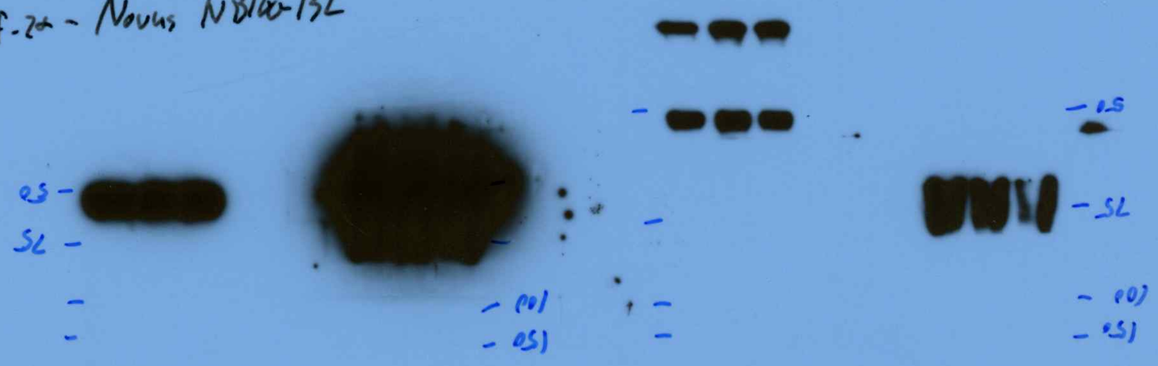

1min

Hep3B - 0,2,8 hrs hypoxia, CoIP
P.D. w/Egr1 ? Egr2   IB: Hif-1α, Hif-2α

IP: Egr2
IB: hHIF2α | hHIFh

AB   Egr1   –   Egr2

Hypoxia (hrs)   0 2 8 | 0 2 8 | 0 2 8

1 2 3 4 5 6 7 8 9

150 –
100 –

α-Hif-1α
1:1000 ON

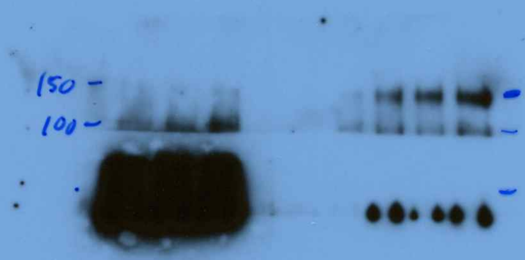

α-Hif-2α
1:1000 ON

90sec

Hep3B cells
0,2,8 hrs hypoxia
WCE
Egr1, Egr2, No AB Pulldown
(Endogenous)

Redo Hif exposure w/Dura

Horse αMouse - HRP
~~GAM~~, 1:3000
1 hr, RT

IP: Egr1, CST 4153
Egr2, abcam ab63943
IB: Hif-1α, BD Bio 610959
Hif-2α, Novus NB100-132

Hep3B - 0,2,8 hrs hypoxia - CoIP
Input - Egr1

5min

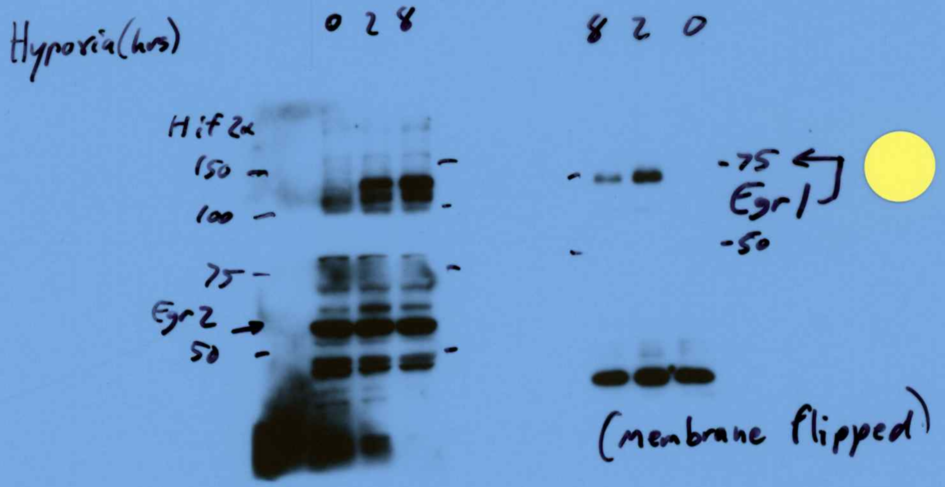

Endogenous CoIP (12-5-10) - Input control

IP: Egr1, Egr2   IB: Hif-1α, Hif-2α

Hif-2α   Novus   NB100-132, 1:1000
Egr1   CST   4154, 1:1000
Egr2   Abcam   ab63943, 1:1000

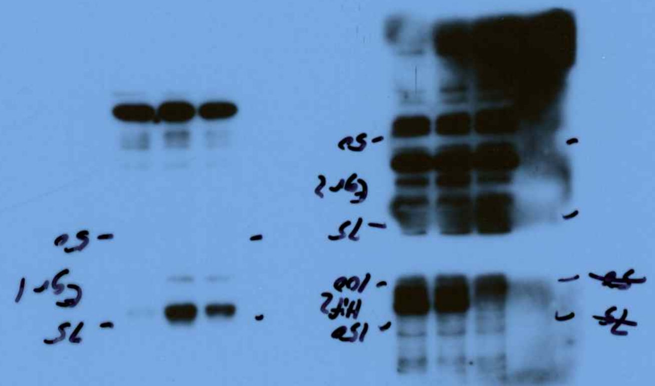

10min

Hep3B - 0, 2, 8 hrs hypoxia CoIP
Input - Egr2

1min

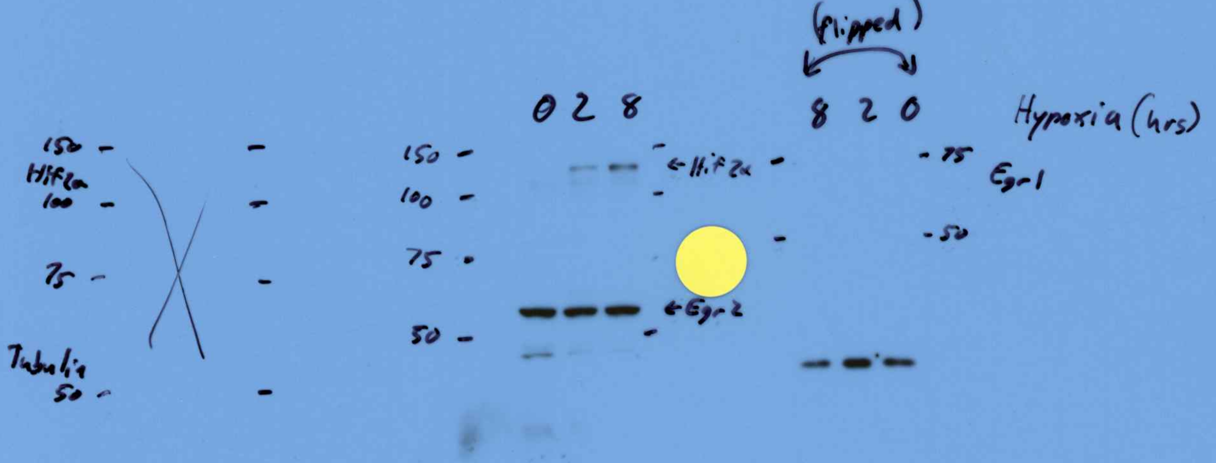

Endogenous Co-IP Input control (12-5-10 exp.)
(IP Egr1, Egr2, W.B. Hif1α, Hif2α)
Strip, re-probe 12-17-10 ctrl blots

Hif-2α } Egr2 paired to 12-17-10 Tubulin #1

Egr1 paired to 12-17-10 tubulin #2

Membrane on left (12-20-10 W.B.) did not stain (improper storage buffer?)

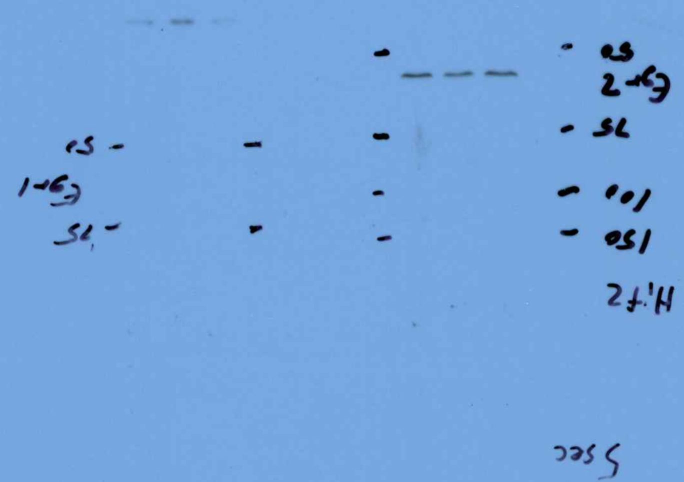

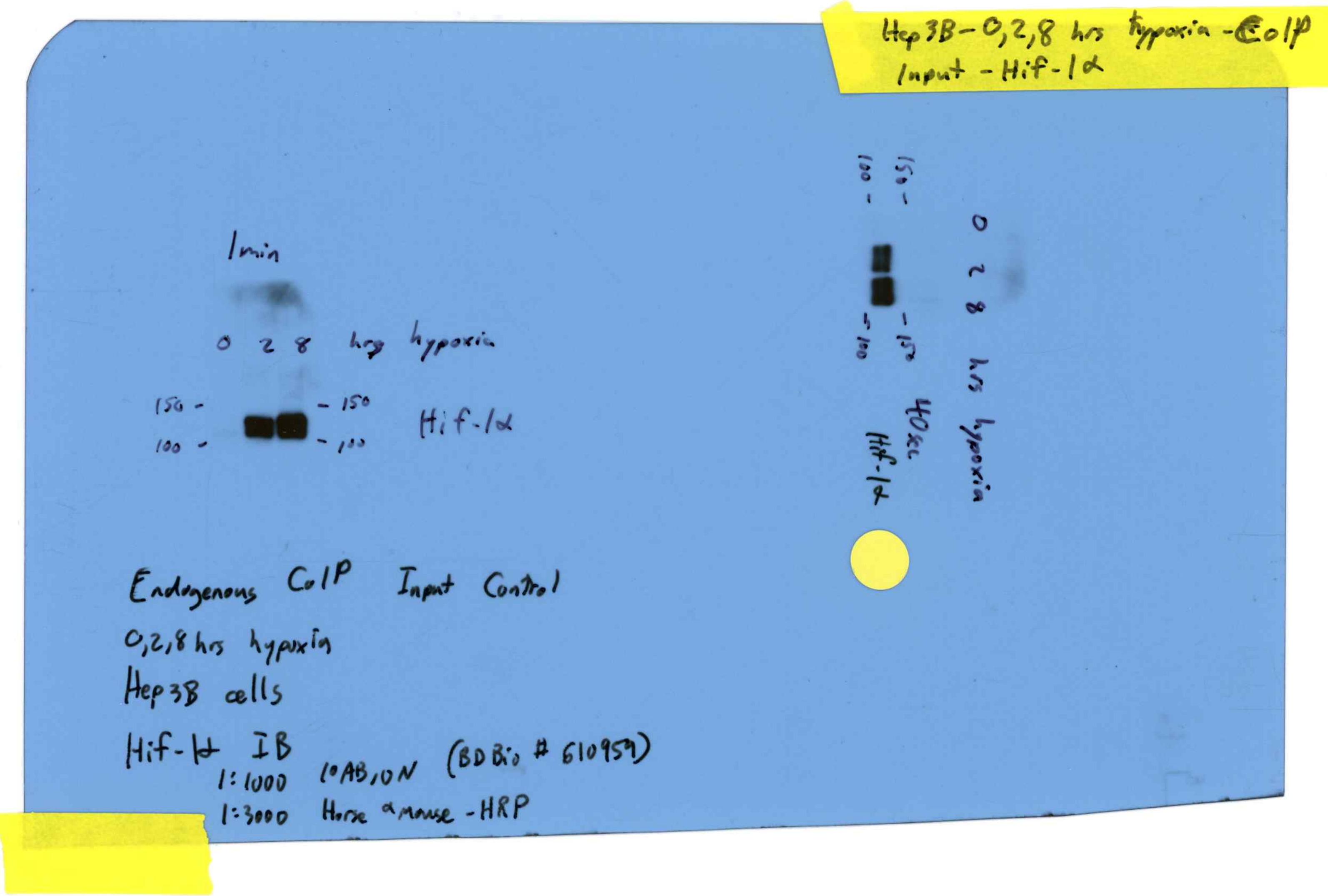
Hep3B - 0,2,8 hrs hypoxia - CoIP
Input - Hif-1α
1min
0 2 8 hrs hypoxia
150 -
100 -
- 150
- 100
Hif-1α
100 -
150 -
0 2 8 hrs hypoxia
40sec
Hif-1α
Endogenous CoIP Input Control
0,2,8 hrs hypoxia
Hep3B cells
Hif-1α IB
1:1000 1°AB, O/N (BD Bio # 610959)
1:3000 Horse αmouse-HRP

Hep3B - 0,2,8 hrs hypoxia CoIP
Input - Hif-2α

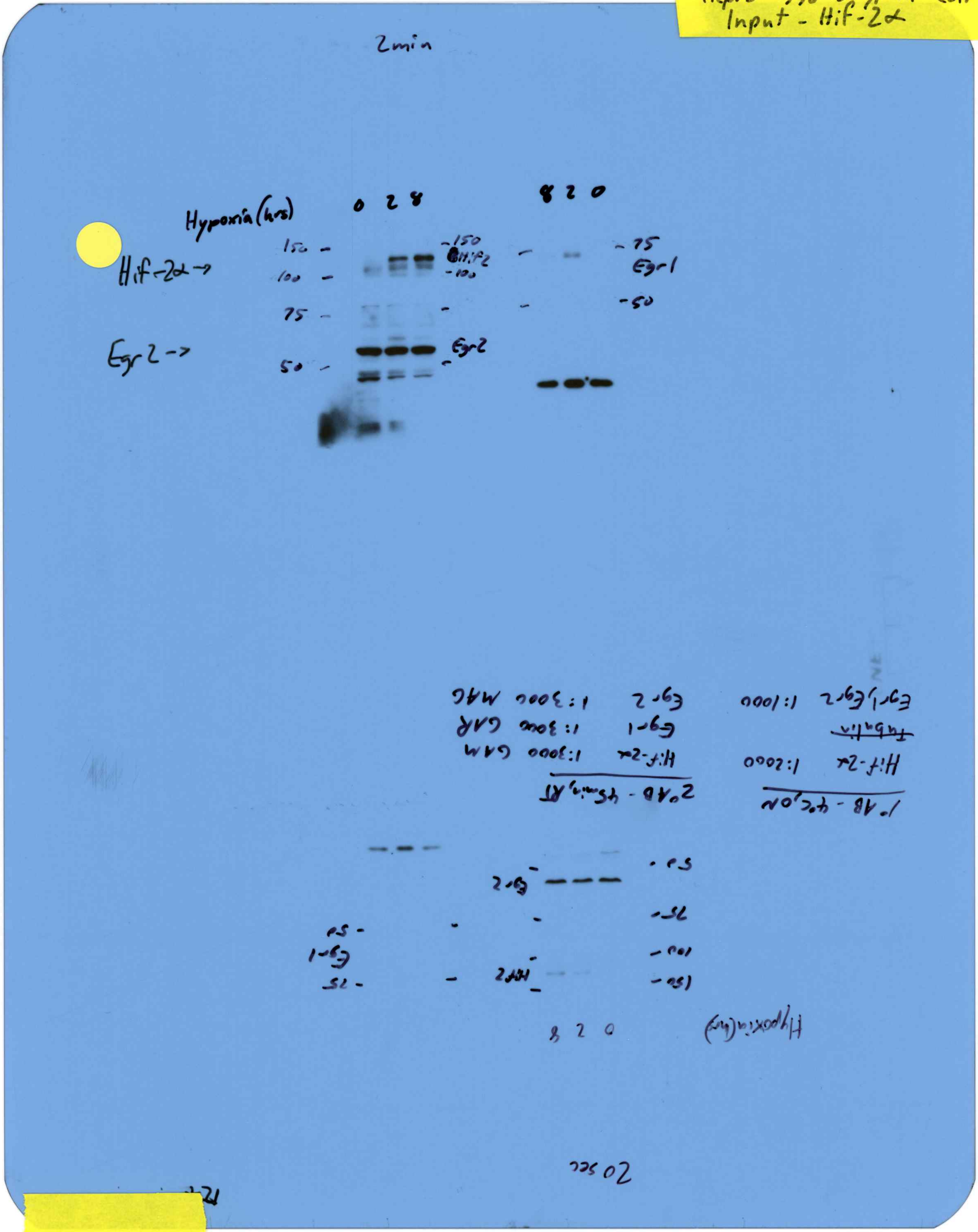

Hep3B - 0,2,8 hrs hypoxia CoIP
Input - tubulin

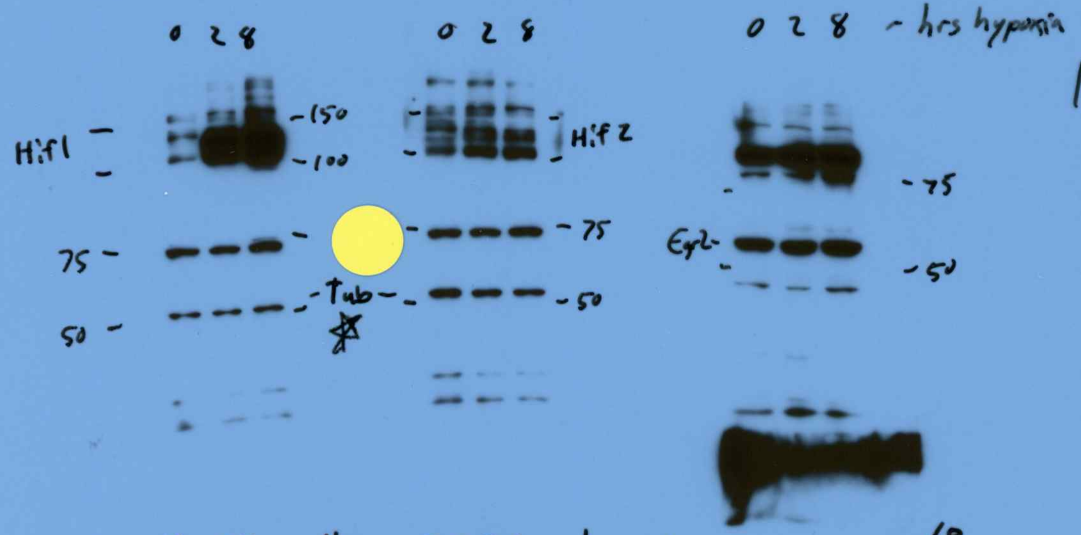

1min exposure

Hep3B cells + 0,2,8 hrs hypoxia
Input Control for Co-IP
20ug WCE/lane

ABs
Hif-1α, BD Bio 610959
Hif-2α, Novus NB100-132
Tubulin, Sigma T9026

1°	2°
1:1000 α Hif1, Hif2, Egr2	1:3000
1:10,000 α-Tubulin	1:10,000
ON, 4°C	45min, RT

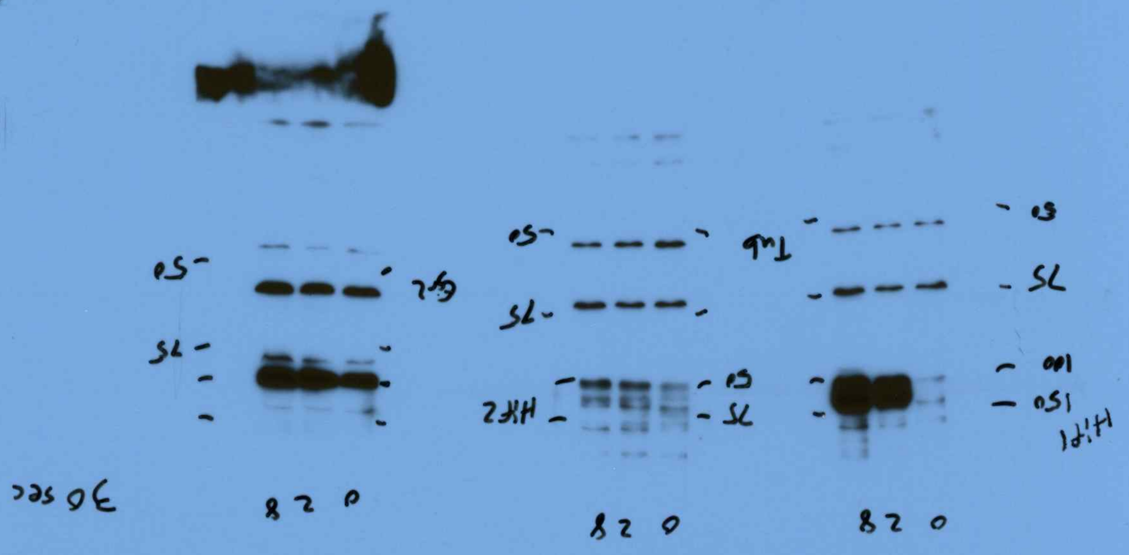

Supplement: Nagati Dioum_JBC_Fig7C_IB scans [file mmc4.pdf]
